# Machine learning analysis of Drosophila testis transcriptomic data reveals potential regulatory sequences

**DOI:** 10.1186/s13040-026-00552-2

**Published:** 2026-03-31

**Authors:** Viktor Vedelek, Balázs Vedelek, Rita Sinka

**Affiliations:** 1 Department of Genetics, University of Szeged, Közép fasor 52, Szeged, 6726 Hungary; 2 Institute of Genetics, HUN-REN Biological Research Centre, Temesvári krt. 62, Szeged, 6726 Hungary

**Keywords:** Testis-specificity, Transcriptomics, Drosophila, Machine learning

## Abstract

**Background:**

The number of accessible transcriptomic data is increasing rapidly due to advances in affordable sequencing technologies. There is also an improvement in the quality and resolution of gene expression maps, which was driven by the advent of single-cell technologies.

**Results:**

Here we present a method, where we integrate transcriptomic data from five different sources, including segmented, single cyst, and single-cell transcriptomic data from Drosophila testis to investigate the expression and accumulation of transcripts in this tissue. The analysis showed that the testis-specific genes have a characteristic profile in the testis, which is predictable using supervised machine learning algorithms (XGBoost). Moreover, dimension reduction with an unsupervised machine learning algorithm (t-SNE) followed by clustering (DBSCAN) of the genes revealed potential regulatory motifs shared by genes in the same group. This approach links the robustness of tissue expression data with the resolution of the single-cell techniques, while masking the weakness of each technique.

**Conclusions:**

The presented approach can be used to find similarly expressed genes and shared regulatory elements or new cell-specific transcripts that could have potential annotation benefits in further research.

**Supplementary Information:**

The online version contains supplementary material available at 10.1186/s13040-026-00552-2.

## Background

The knowledge of gene expression and expression patterns has greatly increased thanks to RNA-sequencing methods and transcriptomic analyses. Transcriptomic data gained from a diverse array of methods covering tissues and cells are available. The advances in the field provided a better understanding of how certain traits are formed through the better understanding of the gene structure, gene expression mechanisms, and the cell and tissue differentiation mechanisms [1–3].

*Drosophila melanogaster* is a fundamental model organism for genetic research; therefore, it is also one of the candidate organisms for large consortial projects, like modENCODE [4, 5]. Furthermore, the popularity of Drosophila led to a vast literature of transcriptomic data and the establishment of tissue and cell expression atlases. Unfortunately, many of the published data from non-consortial backgrounds are not utilized in subsequent research studies. There could be multiple reasons behind this: data requires different processing, needs re-evaluation, newer techniques are better suited, or older methods are obsolete. We aimed to find a means to utilize the processed and published data from a broad range of RNA sequencing experimental findings related to the Drosophila testis. We gathered data from several sources and used statistical methods that gained popularity in the analyses of single-cell sequencing data and machine learning algorithms to further characterize the transcriptome of these sources.

The Drosophila testis makes an excellent model as it is highly complex, yet has distinctive and characteristic features. Spermatogenesis provides a fascinating example of cellular differentiation, where a complex developmental process takes place, resulting in the motile sperm cells [6]. Drosophila testis hosts a wide variety of cell types, including somatic and germ stem cells, mitotic, meiotic cells, and also extremely differentiated cells like spermatids or cyst cells. Germ cell differentiation consists of multiple drastic changes: before meiosis, the spermatocytes exhibit an extreme transcriptional activity, and after meiosis, the gene expression is limited to a few dozen genes [7]. The decrease of transcriptional activity after meiosis during spermatogenesis is conserved, similar to many other features of spermatid development [8, 9]. This is a peculiar phenomenon considering the entire post-meiotic developmental program relies heavily on the gene products synthesized before meiosis in spermatocytes. The differentiation of spermatids includes the formation of various differentiated cell organelles such as the acrosome, axoneme, basal body, and mitochondrial derivatives [10]. The formation of complex new organelles is also accompanied by massive cellular reorganization when the round spermatids elongate up to 1,8 mm. After elongation, the spermatids of the cysts gain their own individual membrane during the individualization process. Individualization is followed by the coiling of sperms and the transfer to the seminal vesicle. During mating the sperms are transferred to the female sperm storage organs, where they remain viable for several days [6]. The complex development of post-meiotic germ cells is fascinating regarding the limited usage of transcription in these stages.

## Methods

### Data source and preparation

Drosophila tissue expression data were collected from FlyAtlas (accessed on 07. 10. 2016.), FlyAtlas2 (accessed on 09. 09. 2021), and ModEncode (accessed on 08. 01. 2019) [5, 11, 12]. Segmented testis transcriptomic data were obtained from Vedelek et al. 2018 [13], single cyst sequencing data from Shi et al. 2020 [14], larval single-cell data from Mahadevaraju et al. 2021 [15], single-cell data from adult testis from Witt et al. 2019 [16], and Fly Cell Atlas [17]. Additional information on data processing is available at Additional Table S1.

A generally used specificity calculation method is the tau score. This score determines if a gene is tissue specific for the whole dataset [18].

$$\:\tau\:=\frac{{\sum\:}_{i=1}^{n}{(1-\frac{{e}_{i}}{\mathrm{m}\mathrm{a}\mathrm{x}({e}_{1},{e}_{2},\:\dots\:,{e}_{n})})}^{2}}{n-1},\:$$

where $\:\tau\:$ is the specificity, *e* is the expression value, *n* is the number of investigated categories sampled from $\:i=1,\dots\:,{n}^{th}$.

The formula of Li et al. 2014 was used to determine tissue specificity (z-score) in each tissue [13, 19], and the specificity of each investigated category of different transcriptomic data.

$$\:{x}_{i}=\frac{{e}_{i}-\stackrel{-}{e}}{\sqrt{\frac{1}{n-1}{\sum\:}_{j=1}^{n}{({e}_{j}-\stackrel{-}{e})}^{2}}},\:$$

where *x* is the specificity, *e* is the expression value, $\:\stackrel{-}{e}$ is the mean expression value and *n* is the number of investigated categories sampled from $\:i=1,\dots\:,{n}^{th}$ and $\:j=1,\dots\:,{n}^{th}$. The scores are available in the Additional Table S1.

After calculation, the z-score testis specificity indices were minmax normalized between − 1 and 1, where 1 represents the highest specificity. The mean value of different tissue expression atlases was used for further analysis. (Additional Table S1)

For the investigation of gene expression patterns, the mean expression values were collected from each transcriptomic dataset, and the specificity indices were calculated. The collected data were normalized and utilized in further investigations.

Data processing and analysis were conducted using Python 3.8. Pandas (1.2.4), NumPy (1.22.4), Scikitlearn (1.3.2), SciPy (1.6.2), XGBoost (2.1.3), and Imblearn (0.12.4) libraries were used for processing data, Seaborn (0.11.1), Bokeh (2.3.2), and Matplotlib (3.3.4) for generating graphs. Dimension reduction method t-distributed stochastic neighbour embedding (t-SNE) and Uniform Manifold Approximation and Projection (UMAP) were used for dimension reduction and visualisation of the data. After testing multiple parameters, random state: 106, perplexity: 100, early exaggeration: 19, number of iterations: 1000 was used to generate t-SNE plot, meanwhile UMAP parameters were as following: random state: 106, number of neighbours: 50. Clustering using density-based spatial clustering of applications with noise (DBSCAN) method was conducted with epsilon values 0.11, 0.12 and minimum cluster values of 40, 60, 90. For XGBoost analyses, the data were randomly divided into training and testing sets at an 80:20 ratio. The entire data consists of 11,019 records; the split resulted in an 8816 training set and a 2203 testing set. Prior to utilizing classification algorithms, the optimization synthetic minority over-sampling technique (SMOTE) was used on the training set to balance the data and avoid overfitting. Each model was optimized with Bayesian search optimization. (Additional Table S1)

Relevant Python codes are deposited at: 10.5281/zenodo.18964638.

### Gene ontology analysis

Gene ontology (GO) analysis was conducted to identify the biological processes, molecular functions, and cellular components associated with the gene set under investigation. For this purpose, PANTHER (Protein ANalysis THrough Evolutionary Relationships) was utilized via the web-based GO enrichment tool [20–22]. The input genes were mapped to the corresponding GO terms using PANTHER’s reference gene set.

### Transcription start sites and motif analysis

Transcription start sites were determined based on transcript annotation, and coordinates were extracted from FlyBase precomputed files. Based on coordinates, genome sequences were extracted from the reference genomes (*Dmel_Release_6*). Extracted sequences were randomly chosen and validated by using BLAST [23]. MEME Suite 5.5.7 was used for motif discovery [24]. MEME algorithm’s basic mode with all motif distribution options (zero or one occurrence per sequence, one occurrence per sequence, and any number of repetitions) was utilized to find 10 motifs with a minimum occurrence of 20 sites in each run. Motifs showing statistical significance (default E-value cut-off) were collected. Using the motif’s position weight matrices, they were mapped to the TSS 1 kb region of gene sets using Biopython’s motif library (1.79) with a relatively permissive 0.8 cut-off.

### Data collection and processing

If a set of genes is regulated by the same transcription factors, their regulatory regions may contain similar motifs, which can result in similar expression patterns. Therefore, we formulated a hypothesis that if we have sufficient transcriptional data of multiple genes with a similar expression profile, we could utilize these for clustering, profiling, and finding regulatory elements and regions that individual groups share. The testis’s complexity, with its variety of cell types and with an extremely high number of expressed genes, makes it ideal to test this hypothesis; thus, we collected data from a variety of sources. To get a general understanding on genes that are expressed predominantly in testes, we collected the tissue transcriptomic data from modENCODE, FlyAtlas, and FlyAtlas2. By using expression values of each tissue, we determined a novel testis specificity index for the Drosophila genes [5, 11, 12]. A tau score and z-score based testis specificity were calculated. Tau score shows the general specificity of genes showing the general overrepresentation, meanwhile the z-score method shows over and underrepresentation of transcripts in testis data. Knowing both the over and underrepresented genes is benfecial in testis, therefore we used the z-score approach in every analysis. Next, we focused on transcriptomic data gained only from the testis. We collected five different datasets: RNA seq from segmented testis (3 stages), single cyst sequencing (8 categories), single-cell sequencing from larval testis (10 cell types), single-cell sequencing from adult (9 cell types), and the data from the fly cell atlas (39 cell types) [13–17]. Data from these sources has different benefits and drawbacks. The experiment with segmented dissection of the testis is robust, yielding plenty of reads; however, the origin of these reads cannot be traced back precisely to the cell types of the segments, therefore, the resolution of such experiments is low. Single-cell experiments, on the other hand, yield fewer reads, the coverage is lower (less robust), but their resolution is high, as the origin of the reads can be traced back to single-cells. Investigating these data, we first extracted the mean expression values of each gene in all of the originally annotated categories and kept them as a separate dimension for all datasets. In the case of Fly Cell Atlas data, first, the mean value of each annotated cell type was collected from the loom files to represent the given cell type (Additional Table S1). Next, we applied the specificity calculations (z-score) for each dataset and kept every sequenced category. This way, a single dimension represents expression values for every source, and the specificity of expression is represented in every discussed dimension. As a result, experiments with higher resolution get more representation, with more dimensions, yet the expression values have a limited effect on further analyses, as they are restricted to a single dimension. This also means that the expression values of methods with more limited resolution have proportionately greater impact, which is beneficial due to their robustness. Prior to any other analyses, we used standard score normalization (z-score standardisation) on the full dataset (Additional Table S1).

## Results

### Unsupervised machine learning shows distinctive regions with testis-enriched genes

**Fig. 1:**
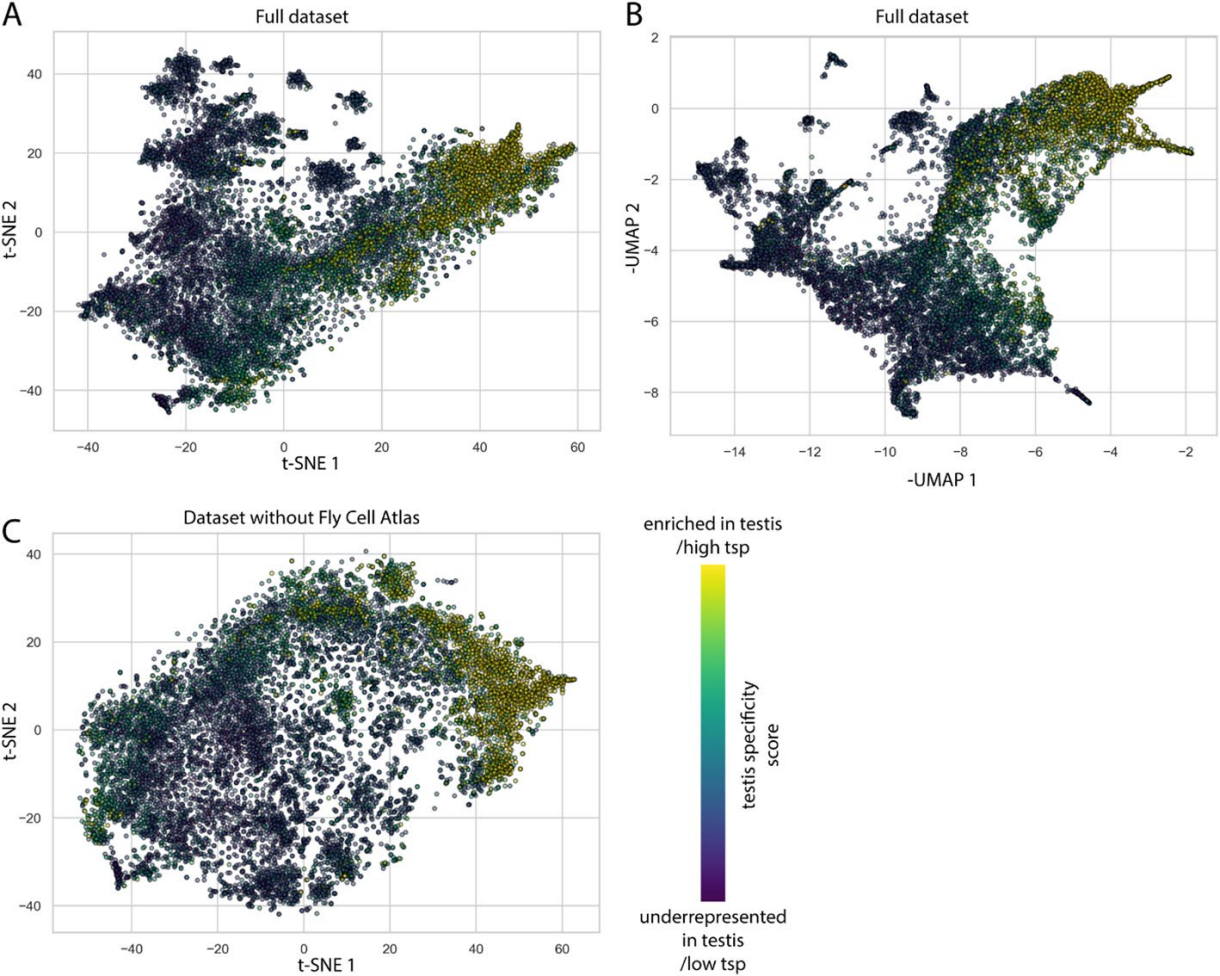
Dimension reduction of the testis transcriptome dataset. Dimension reduction methods coloured according to the testis specificity (tsp) show the distribution of genes on the full dataset after (**A**) t-SNE and (**B**) UMAP or (**C**) t-SNE analysis without single-cell data

We utilized two unsupervised machine learning methods, the t-distributed stochastic neighbour embedding (t-SNE) analyses and Uniform Manifold Approximation and Projection (UMAP) for dimension reduction of the gene dataset [25, 26]. Both of the approaches give similar results (Fig. 1A, B), but since t-SNE produced more visually distinctive groups, we used t-SNE for further analyses. Testis-specificity was previously calculated based on three tissue expression atlases (FlyAtlas, FlyAtlas2, ModEncode) to determine which genes are characteristic of this tissue [5, 11, 12]. Specificity indices take a value between − 1 and 1, where 1 represents genes solely expressed in the testis. The specificity indices and tissue expression data were not utilised in either dimension reduction method; however, testis-specific genes show enrichment in certain regions (Fig. 1).

We were interested in the effects of the integration of multiple datasets. The highest resolution was provided in the Fly Cell Atlas data; therefore, we investigated what differences would occur if this dataset were left out of the t-SNE analysis (Fig. 1C). Additionally, the t-SNE results of the whole dataset were compared to the incomplete dataset missing the Fly Cell Atlas data (Additional file S1). We could draw two conclusions: one is that the addition of the fly cell atlas data greatly decreases the noise with the establishment of new groups. The decrease in noise could make clustering more precise. The other observation is that the existing groups are not affected considerably by the additional data. The presence of stable groups is promising, as different methods strengthen their existence. We considered these properties beneficial for further investigations as they emphasize the advantages of using additional datasets.

### XGBoost is efficient in classifying testis-enriched genes

**Fig. 2:**
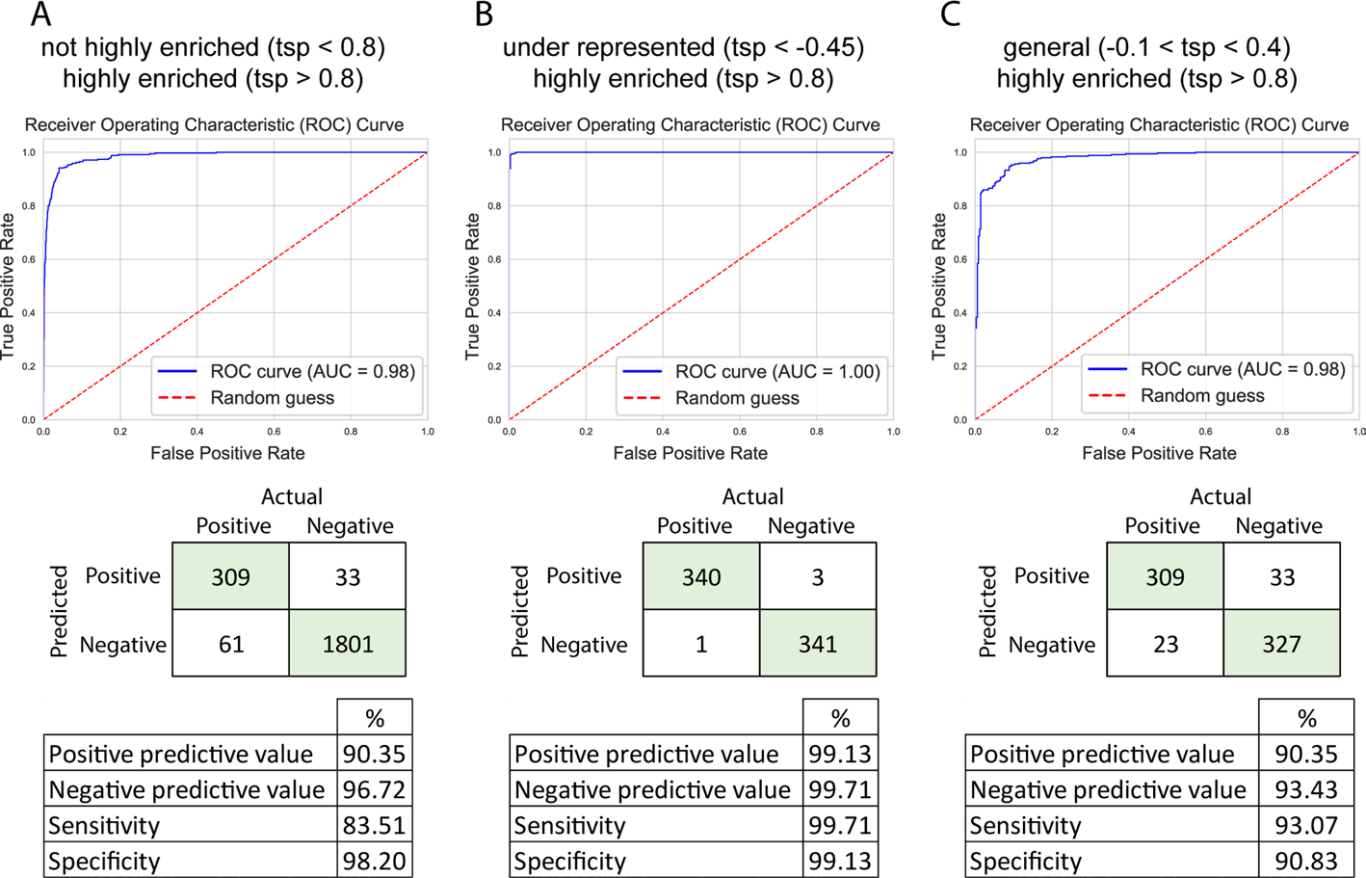
Performance metrics of XGBoost models on specificity categorisation. Receiver operator curves (ROC) are indicated on the graph with blue line, meanwhile, theoretical random guesses with a red dotted line. Confusion matrices indicate the performance of the model on the test data. The associated predictive values, sensitivity and specificity are presented in the tables. (**A**) The chart shows the model that investigated highly testis-enriched genes, with not highly enriched ones. (**B**) The model compared genes that are underrepresented in the testis with highly enriched genes. (**C**) The model utilized generally expressed genes compared to highly testis-enriched ones

Although testis-specificity was not used for t-SNE calculations, the mapping of testis-specificity to the t-SNE map revealed distinctive regions, with genes that show lower or higher testis-specificity (Fig. 1A). It is important to emphasize that the testis-specificity was determined using entirely independent data from that utilized for the training of the models. The presence of regions where testis-specificity is enriched raised the question of whether testis specificity could be determined using exclusively expression data from the testis? To test this, we utilised the extreme gradient boosting (XGBoost) classification algorithm. We considered genes that have at least a 0.8 testis specificity index as testis-specific, dividing the genes into two groups (> 0.8 and < 0.8). Prior to applying the model, we normalized the data and utilized the synthetic minority over-sampling technique (SMOTE) in order to avoid overfitting. For the evaluation of the prediction model, the receiver operator curve and area under the curve (ROC/AUC) were utilised. A model is performing better if the area under the curve (AUC) score is close to 1, and it is making random guesses if the score is close to 0.5. We found that the model gives relatively good scores, as we obtained a 0.98 AUC score. Meanwhile, other metrics were also satisfactory: specificity 98.2%, sensitivity 83.5%, positive predictive value 90.3%, and negative predictive value 96.72% (Fig. 2A). Next, we were interested in how the model performs if there is a more distinct difference present in the testis specificity indices. We established two datasets: one set contained the underrepresented genes that have low testis specificity values (<-0.45), these genes have generally low expression values in the testis compared to expression in other tissues. Another set where generally expressed genes were collected (-0.1<, < 0.4), these genes have a similar expression pattern across different tissues. These datasets were compared with the set containing the highly testis-enriched ones (> 0.8). These models performed even better (Fig. 2B, C), suggesting the presence of a characteristic profile of these groups. The model that differentiates between the testis-specific and underrepresented genes in the testis has an AUC score of 0.9995, and all the other metrics are above 99%. The model comparing the generally expressed genes with the testis-enriched ones showed an AUC of 0.98 and a score above 90% in every other metric.

XGBoost is a decision tree-based method that allows us to easily collect feature importances that represent how the splits, the divisions of the data on the branches of the tree, were performed in the model. This gives insight into the working of the model and helps to interpret the data. Feature importances from the XGBoost results imply which characteristics have a higher impact on determining the classifications. Three types of feature importance can be extracted from the models: gain (feature contribution to model performance), weight (feature representation in splits), and cover (feature coverage across all splits) (Additional file S2, Additional Table S2).

The 3 models show high gain from spermatocyte data, mainly from middle and late stages. The model that classifies generally expressed and highly testis-enriched gene expressions also shows high gain in the elongating spermatids. Higher scores of weight and cover similarly represent the above-mentioned features: spermatocyte, spermatid data, and overall expression means. Based on these results, testis-specific genes are enriched in mature spermatocytes and spermatids. This observation is not surprising as the spermatogenic process relies heavily on the gene products expressed in spermatocytes. Several factors were identified contributing to gene expression in spermatocytes; there are known testis-specific transcription factors and complexes that govern the gene expression in these stages [27–30].

### Transcription factor association with XGBoost shows low positive prediction value and sensitivity

**Fig. 3:**
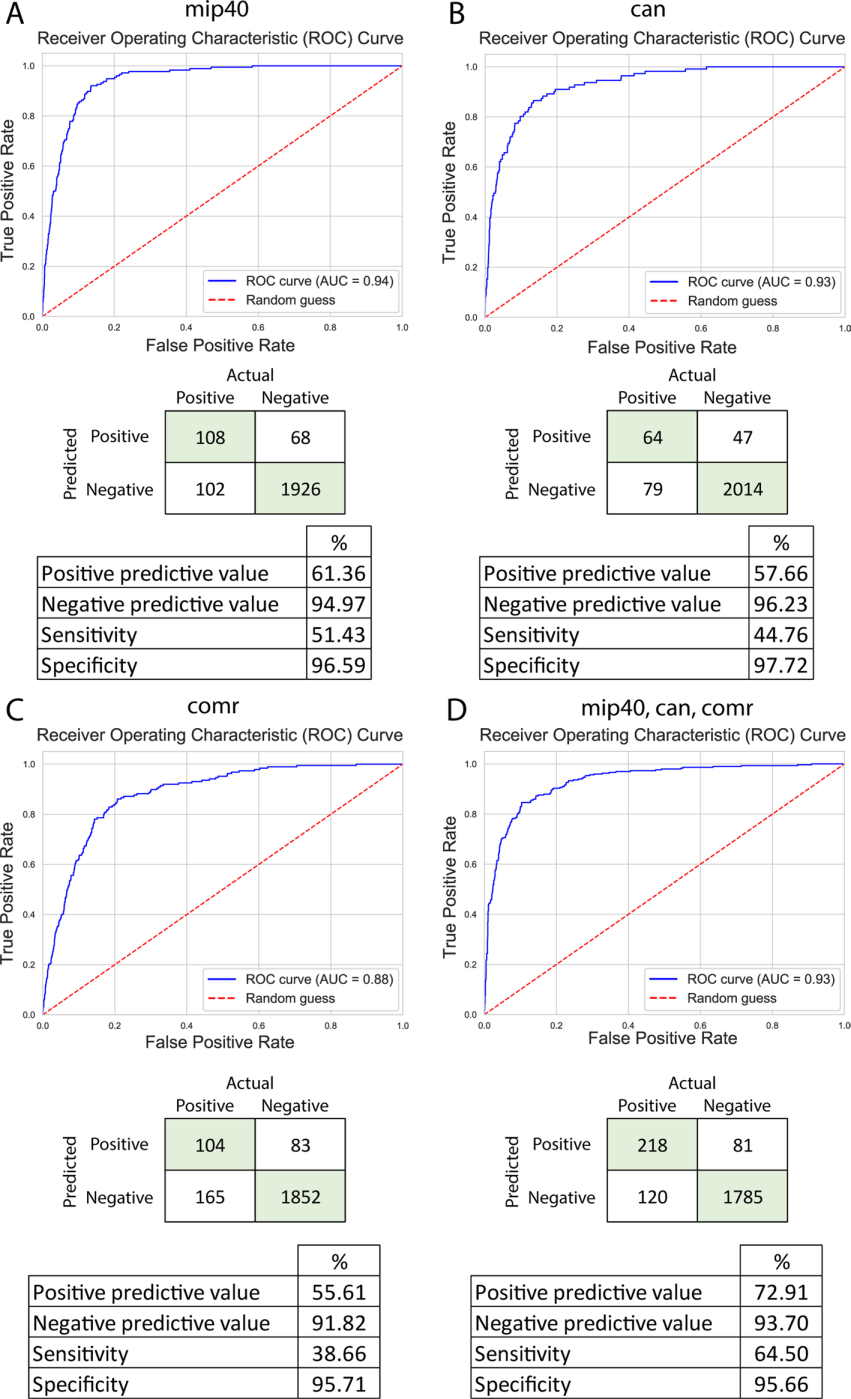
Performance metrics of XGBoost models on specificity categorisation. ROC curves are indicated on the graph with a blue line; meanwhile, theoretical random guesses are shown with a red dotted line. Confusion matrices indicate the performance of the model on the test data. The associated predictive values, sensitivity, and specificity are presented in the tables. The model (**A**) represents mip40 predictions, (**B**) can predictions, (**C**) comr predictions, and (**D**) predictions of all three categories

We used XGBoost to test whether the experimental results about testis-specific transcriptional factors from Laktionov et al. can be used for classification [27]. The testis-specific transcription factor binding sites of three different complexes (tMAC, tTAF, MMB/dREAM) were experimentally identified using DamID. We investigated whether genes associated with these sites could be classified using the transcriptomic dataset. The Myb-interacting protein 40 (mip40 – tMAC activator, MMB/dREAM repressor), cannonball (can – tTAF activator), and cookie monster (comr – tMAC activator) were tested using direct and indirect target genes. Additionally, we established a gene set where every target genes of the transcription factors were included. Since the number of genes with binding site associations was relatively small, we used SMOTE to balance the data. The XGBoost models provided an AUC score of 0.94, 0.93, 0.88, 0.93 for mip40, can, comr, and all three targets, respectively (Fig. 3).

Despite the higher AUC scores, these models have a relatively low positive predictive value and sensitivity. The best positive predictive value (72.91%) was achieved when we investigated all three transcription factor-related gene lists together. Based on this, we hypothesize that the low number of target genes is the reason why models are not performing well compared to testis specificity values. All four models have the highest gain from the late primary spermatocyte feature. The model that classifies cannonball targets has additional high gain from the mean expression and the expression of the tip of the segmented testis (Additional Table S2, Additional file S3).

The model that classifies all three target genes has additional gain from other spermatocyte categories, making it similar to the testis-specific classification. Regarding weight and cover, segmented testis tip, single cyst sequencing data of late cysts, and spermatocyte features were represented in the models.

Meanwhile, testis specificity can be predicted with high accuracy; genes with testis-specific transcription factor associations were predicted relatively poorly.

### The testis-specific transcription factor associated genes show considerable overlap with the testis-enriched genes on the t-SNE plot

**Fig. 4:**
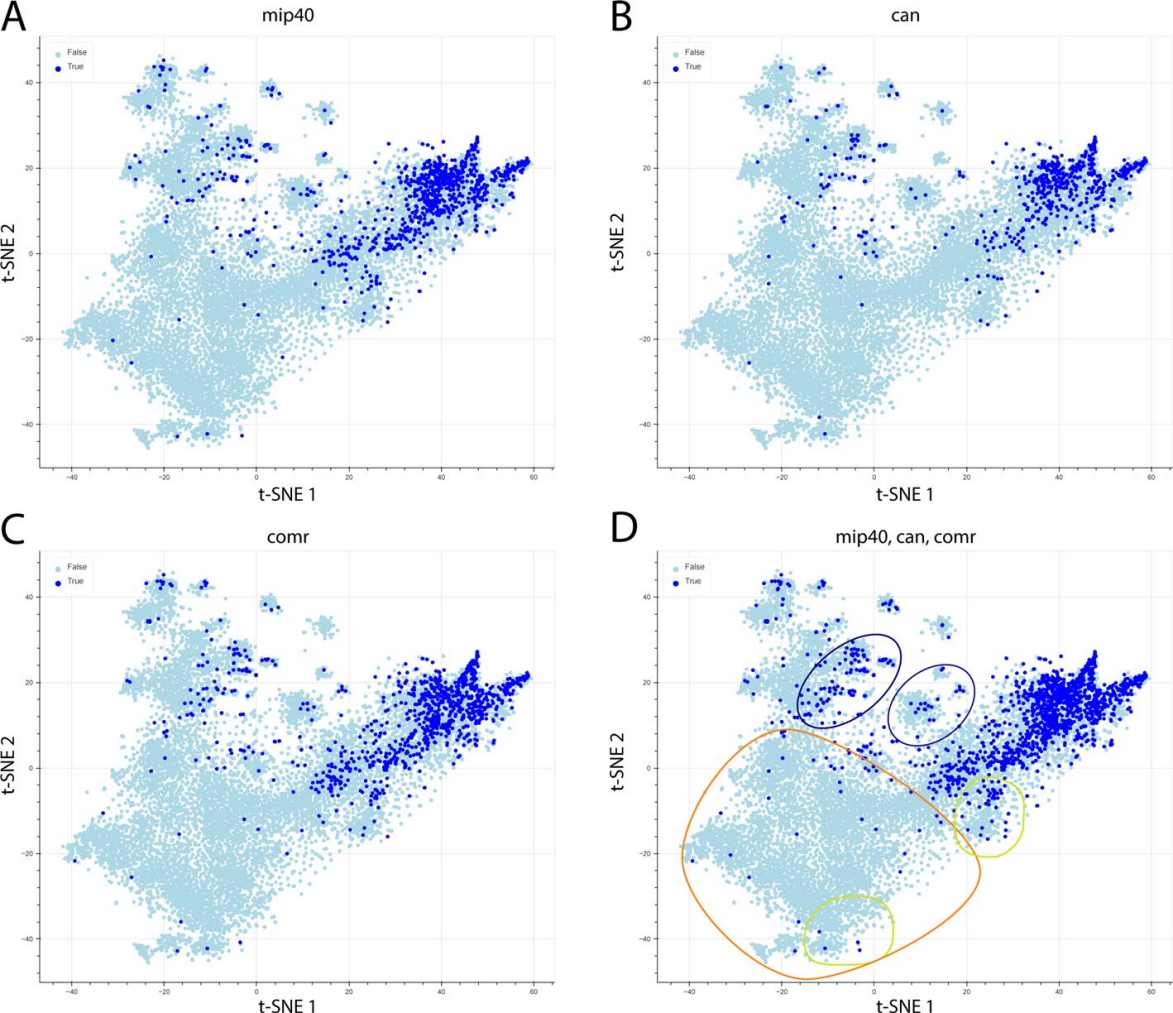
t-SNE plot highlighting the genes associated with testis-specific transcription factors. (**A**) shows mip40 associated genes, (**B)** can associated genes, (**C**) comr associated genes and (**D**) mip40, can and comr associated genes in dark blue. Light green highlight on regions that contain a higher number of testis-enriched genes but fewer associations with testis-specific transcription factors. The orange highlight shows a region where few transcription factor associations are present. Purple highlight shows regions with a lower number of testis-enriched genes, but with testis-specific transcription factor associations

The genes associated with mip40, can, comr, and all three targets were plotted to the t-SNE map (Fig. 4).

The distribution of these genes was similar and showed a great overlap with the testis-enriched genes on the right side of the t-SNE plot (Fig. 1A). Interestingly, not all of the testis-enriched regions are showing a high number of associations to the investigated factors, and some regions with rather low testis specificity show multiple associations. (Fig. 4D) A trend can be observed; the region in the negative t-SNE values shows minimal associations, while most of the associations are in the positive t-SNE values of both axes.

### Clustering the t-SNE data with a consecutive density-based spatial clustering of applications with noise (DBSCAN) analysis

Next, we shifted our focus to the genes that are positioned closely on the t-SNE map. Dimension reduction methods are extensively used to visualize data from single-cell sequencing results and to formulate groups from cells with similar transcriptomes. Similarly, the t-SNE map presented here can be investigated further with a consecutive density-based spatial clustering of applications with noise (DBSCAN) analyses as an attempt to identify the similarly expressed genes. By using multiple parameters for DBSCAN, the size of groups can be changed, allowing us to determine clusters with different sizes and even subgroups (Additional file S4).

### Genes in DBSCAN groups show cell type and gene ontology enrichments

**Fig. 5:**
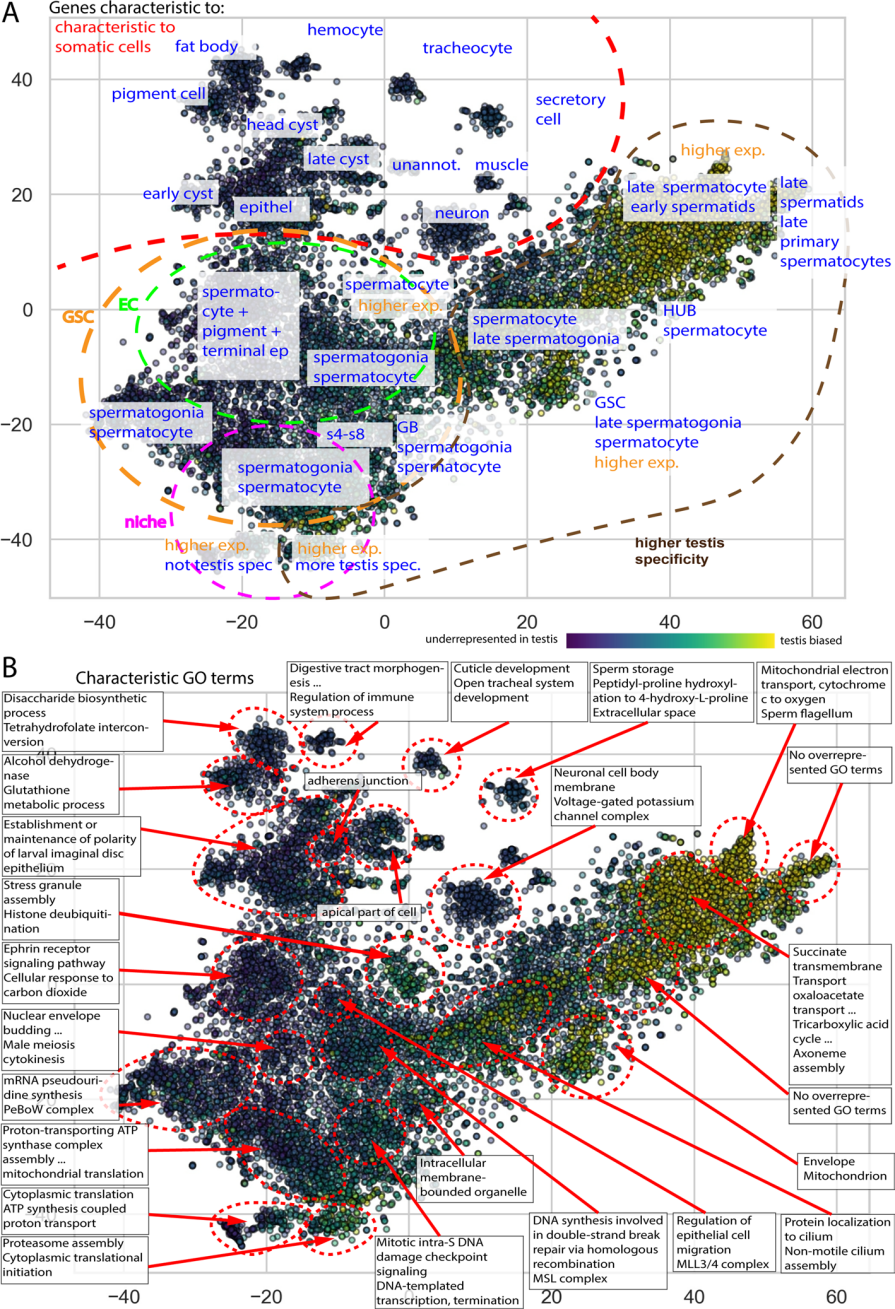
Characteristics of groups established by DBSCAN. Figure 1A extended with annotations of (**A**) profiles and regions based on input dimensions, with additional comments on expression and specificity. (**B**) Fig. 1A annotated with the most characteristic GO terms of the given regions

We present a variety of different results for the DBSCAN analysis (Additional File S4). The established groups were tested based on their expression profiles, which helped us to determine groups with specific expressions (Fig. 5A, Additional File S4).

Interestingly, a smaller cluster of genes could be associated with certain somatic cell types, suggesting that those genes are characteristic but not necessarily exclusive to these cells. The large central cluster can be subdivided into multiple areas. On the left, generally expressed genes are present within a variety of cell types; meanwhile, on the right end, more differentiated germ cell-associated genes are present. Next to determining the cell types characteristic of clusters, gene ontology analyses can also be concluded, which gives insights into the functionality of genes in the given cluster. Therefore, we selected a subset of the groups established by DBSCAN for further investigation (Fig. 5B, Additional Table S3-S5). Using Panther for GO analyses on these clusters, we can determine potentially interesting groups, as we can see the genes characteristic of germ and somatic cells can be characterized by different positions on the t-SNE map. From the analyses, we produced a simplified map for visualisation (Fig. 5B), meanwhile, detailed data is available in the supplementary material (Additional Table S3-S5).

Genes that have elevated expression in neurons cluster together and are associated with neuronal GO terms and pathways. The genes expressed in epithelial and cyst cells are mapped closely; nevertheless, they can be distinguished from each other (Fig. 5). The genes enriched in early cyst cells and epithelial cells can be grouped into which group the “epithelial development”, “chemotaxis and polarity” GO terms were also enriched. This suggests that cyst cells and epithelial cells share a gene set that is characteristic of both cell types. The genes related to differentiated cyst cells differ from each other and from the epithelia.

Genes that are more characteristic to the germ line spread wider on the t-SNE map, with several distinctive groups of genes (Fig. 5). These genes could not be strictly associated to a certain cell type, yet they represent distinct developmental stages. Groups that associate with later developmental stages have higher testis specificity than groups associated with earlier stages; however, the determination of strong enrichment of a particular developmental stage is not obvious. In contrast, GO enrichment reveals distinguishable functional differences in these groups (Fig. 5B, Additional Table S3-S5). On the left side (*x* < 0) of the TSNE map, a lower number of testis-specific genes are present; in these regions, GO term enrichments show terms that can be associated to standard cellular functions, such as DNA, RNA and ribosome-related processes, transcription, translation, and cytokinesis. On the right side (~ x > 0), where testis-specific genes are enriched, more granular GO terms occur, such as terms related to cilia, axoneme, and flagellum. Additionally, metabolism-related terms are also represented in this region. This is not surprising as a high number of metabolic genes have testis-specific paralogs [13, 31]. These observed patterns of GO enrichment are in line with the cell type enrichments and can be associated with the known details of spermatogenesis (Fig. 5). The genes associated with the testis-specific transcription factors are mostly present in the regions where more differentiated GO terms were present, meanwhile, underrepresented in the regions with more general terms (Figs. 4 and 5).

### DBSCAN clusters can be used to identify DNA motifs

**Fig. 6:**
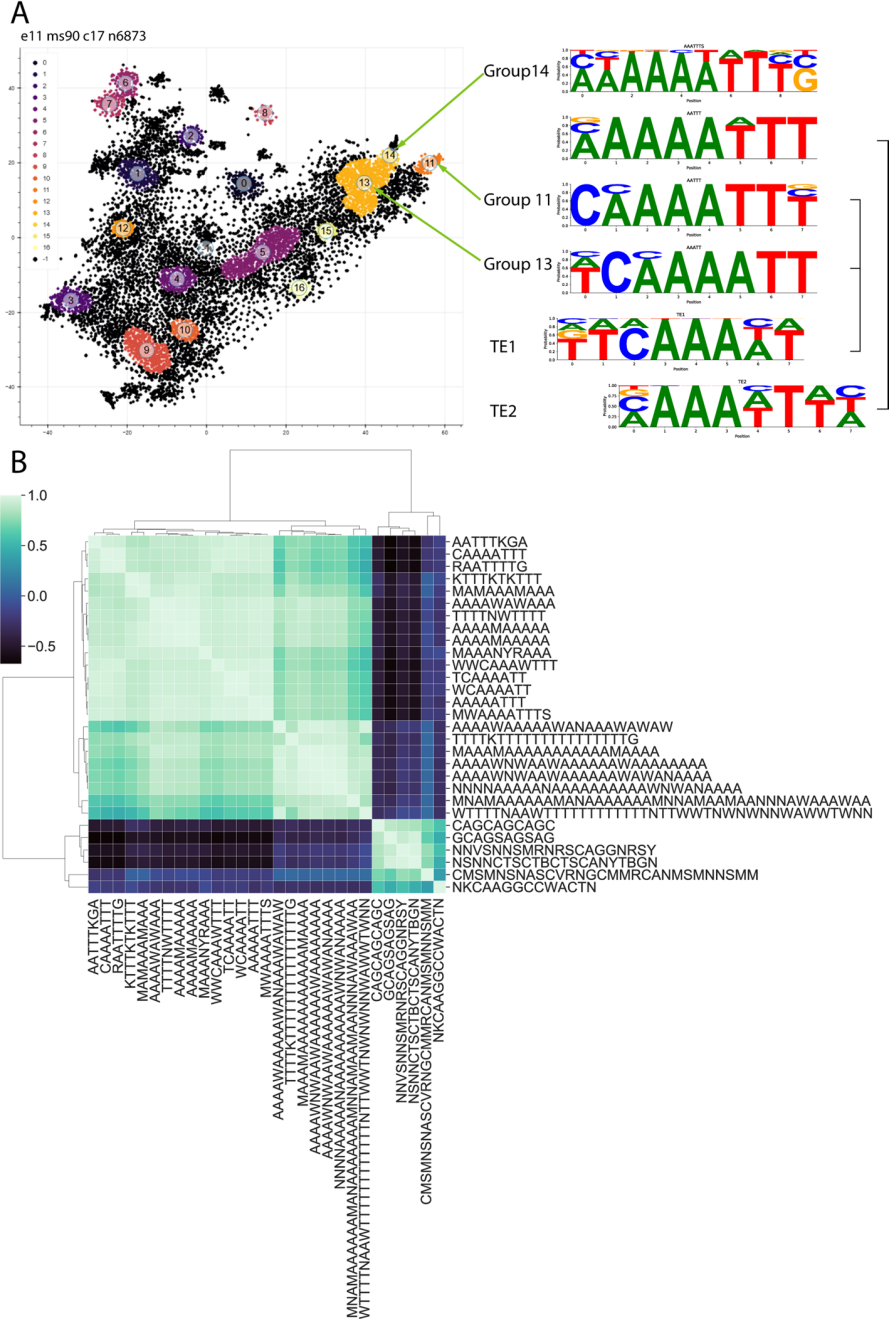
Motifs of the 5’UTRs. (**A**) Motifs that are similar to the previously identified TE1 and TE2 elements (TCE component motifs) from group 11, 13 and 14. (**B)** Correlation of the discovered motif’s positions in the TSS region. Only genes that have an expression value greater than 1 were used for motif discovery and mapping

A motif of the 5’ region of multiple testis-specific genes is described in the regulation of testis-specific expression: the translational control element (TCE), which is present in the core promoter and 5’ untranslated region (UTR) of certain testis-specific transcripts [32]. Originally, two motifs were identified (TE1 and TE2) from testis-enriched genes, but given the similarity of these two motifs, they were merged into a single one, and the TCE motif was established [32]. We investigated whether we could find the TCE element in the 5’UTR RNA sequence of genes from the groups with the highest testis specificity. We collected UTR sequences from highly testis-enriched group 11, 13, and 14 (DBSCAN with e-11 ms-90) results and used these sequences for motif discovery in the Meme suite (Fig. 6). We successfully identified several motifs using these sequences; moreover, we found motifs that show similarity with the TCE motif (Fig. 6A, Additional file S5).

The motifs found in these groups, although they resemble the TCE motif, have differences. The core of the original TCE contains an AAAN sequence; meanwhile, our results show AAAA. Interestingly, the motifs from groups 11 and 14 were also different, and the motif of group 14 was more similar to the original TE2 regarding the absence of a high ratio of C at the front of the motif. Meanwhile, the more complex motif from group 11 showed more similarity to TE1 and the whole TCE (Fig. 6). More interestingly, this motif also shows high similarity to the testis-specific TAF binding motif (CAAAATTY) [33]. Our data suggests that the original TE1 and TE2 might be two different motifs after all, which are slightly different yet characteristic of distinct groups of testis-enriched genes. With this analysis, we also identified other enriched motifs (Additional file S5). Motifs found could be distributed to 3 main types: 8–12 nt AT rich motifs, ~ 25 nt AT rich motifs, and motifs with higher complexity (Fig. 6B). We hypothesize if a group of genes have similar expression pattern in testis, they could also have similar regulation, which ensures the appropriate expression profile. Next, the identified motifs were mapped to the 1 kb range of the transcriptional start sites (TSS) of the genes. A permissive 80% matching was used for mapping on filtered sequences. Gene sets were filtered based on expression profile (1<, 100<, 1000<), testis-specificity (> 0.9, 0.5>, < 0, <-0.5), and gene lists associated with testis-specific transcription factors (mip40, can, comr) were also tested. A cut-off of gene expression was set to at least 1, to filter minimally expressed genes (Additional file S5).

**Fig. 7:**
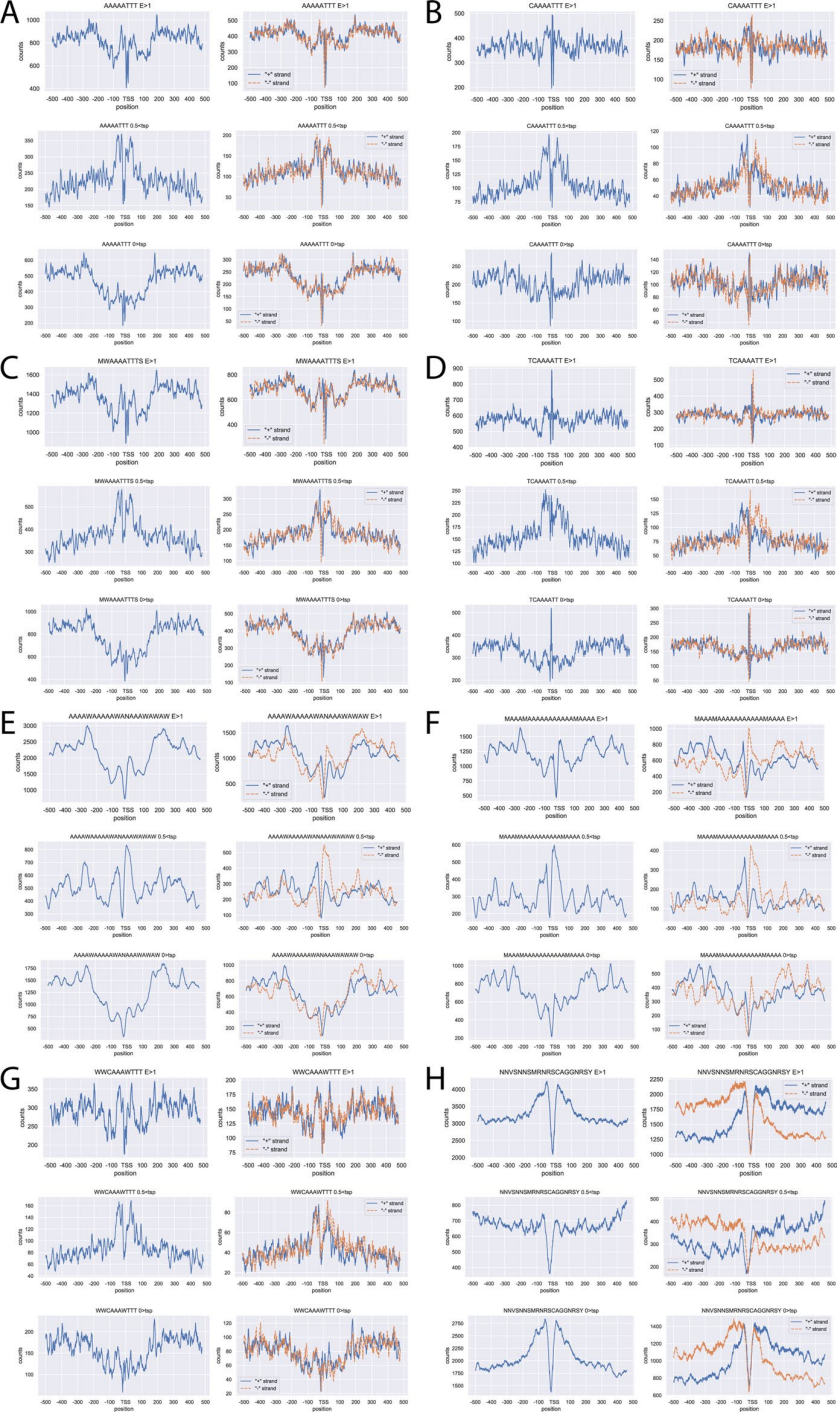
Representative patterns of different motifs around TSS region. Diagrams are paired; the first diagram shows the overall distribution, the second shows a breakdown based on the two DNA strands. Chart titles contain additional information on the motif and filter used: E > 1 - expression is greater than 1, 0.5 < tsp - testis-specificity index is greater than 0.5, 0 > tsp - testis-specificity index is less than 0. **A-D** represent the TCE-like motif patterns. **E**,** F** represent the patterns of longer A/T rich motifs. G represents a motif without differences based on strand, and H represents a motif with strand association upstream and downstream of TSS. Additional graphs on identified motifs are available in Additional file S5

The results showed that most of the motifs are not just enriched downstream but upstream of the TSS (Fig. 7, Additional file S5).

Investigating the distribution patterns, we can find considerable differences as the expression gets higher. Highly expressed genes show similar patterns to genes with high testis specificity. This is not surprising as highly testis-specific genes often have high expression [13]. The patterns observable with the three transcriptional factors (mip40, can and comr) often differ from the general pattern and are more similar to the testis-enriched patterns. By applying a filter for testis specificity, the majority of the motifs’ patterns exhibit considerable variations; the peaks found in more specific categories are absent in less specific groups. In summary, the most promising motifs show a difference between the general, high, and low specificity groups. The TCE-like motifs show very similar patterns to each other (Fig. 7A-D), the peaks present in the higher testis specificity group are not present in the lower testis specificity groups. These motifs are slightly strand-specific; in the case of oligo A/T rich motifs, the motif position is often strand-dependent. This results in different patterns in a strand-specific manner before and after the TSS (Fig. 7E-F). Some motifs show no strand dependence (Fig. 7G), meanwhile some motifs show a considerable swap in occurrence between the strands (Fig. 7H). The enrichment of most of these motifs in testis-enriched genes’ TSS regions and the lack of them in underrepresented genes’ TSS regions suggests the importance of these motifs in testis-specific gene expression. Nevertheless, further studies are needed to clarify if they provide a DNA structure that makes TSS accessible or serve as a platform for protein interaction partners. We believe that these results prove that our initial hypothesis is correct, and a systematic screening for further motifs could contribute to getting better insight into sequence-specific regulation in male germ cells.

## Discussion

In this investigation, we analyzed multiple facets of gene expression in the testis by utilizing an extensive range of transcriptomic data (Additional file S6). We refined the testis-specificity index, which we used previously to determine the specificity of genes [13]. Now it reflects data from three different expression atlases (ModEncode, FlyAtlas, FlyAtlas2), and is scaled to have theoretical values between − 1 and 1. The new testis-specificity score is more accurate, and it is easier to interpret.

We have no knowledge of where previously t-SNE with DBSCAN was utilised on gene features for clustering. By the usage of t-SNE additional information can be obtained on gene expression patterns. Five different sources of gene expression data (RNA seq from segmented testis, single cyst sequencing, single-cell sequencing of larval testis, single-cell sequencing of testis, fly cell atlas data) were integrated with the investigation of the benefits of extending the datasets [13–17]. The results showed that, in Drosophila testis, the testis-specific genes have multiple unique expression profiles, associated mainly with the germline. XGBoost machine learning models revealed that gene’s specificity can be derived with a good approximation solely from their expression pattern in the testis. This feature is associated with the fact that genes enriched in the testis generally show elevated expression and possess distinct expression patterns in a select group of dedicated cells. This seems trivial, yet previous analysis showed that highly enriched transcripts do not necessarily have high expression in the given tissues [34]. The dimension reduction with t-SNE showed that highly testis-enriched genes are not forming a single, but multiple clusters, suggesting their expression pattern and therefore their regulation could be different between the clusters. The clustered genes can be associated with earlier or later developmental steps, but to no exact stage. Additionally, different GO enrichments are observable in the clusters, implying a functional aspect of clustering and the co-regulation of processes and pathways. This is in line with the expectations, as consecutive spermatid developmental steps are continuous, yet have considerable different characteristics [6, 10]. However, identified groups and associated genes and GO terms are needed to be addressed with care. Groups established are showing similar features, yet genes of a group could have very different attributes. For example, there are highly testis specific genes present in groups that are characterised by low testis specificity. Since the t-SNE map does not represent cells, but genes, the cell type associations need to be considered as a property of the gene sets. Nevertheless, the members of each group was selected based on similarities in expression patterns and GO terms could be associated to these groups suggesting the presence of a functional clustering.

Regulatory elements could also be investigated using the genes in groups. Investigating 5’UTR regions, TCE element variants were identified in different groups [32]. The presence of the variants in different groups raises the possibility that these variants could have an impact on the accumulation of the transcripts; therefore, the previously identified TCE-related motifs, TE1 and TE2 might have slightly different functions. Another interesting observation on the t-SNE map is the high number of mip40, can, and comr independent testis-enriched genes [27]. Genes associated with mip40, can, and comr were predicted poorly by machine learning algorithms; however, as expected, they could be associated with some of the motifs that were found based on the highly testis-specific groups. The highly testis-enriched genes’ 5’ UTR regions were used to find additional motifs; these motifs could be mapped upstream and downstream to the transcriptional start site (TSS). The general pattern shows an enrichment of the motifs before and after the TSS. The identified motifs tend to have a high occurrence in testis enriched genes, showing different patterns compared to not testis enriched genes when they are mapped to the TSS region. The presented patterns show difference between highly testis enriched and underrepresented genes, suggesting that they are characteristic to the enriched genes. In the case A or T rich motifs a strand dependent manner is observable. The presence of AT rich motifs are not surprising, as testis-enriched genes tend to have a higher AT content around their TSS and could contribute to the accessibility of the promoter region [35–38]. The results showed that the systematic screening for motifs could contribute to getting better insight of sequence specific regulation in spermatids. The future testing of these motifs with transgenic reporter constructs could provide validation and additional useful insights.

## Conclusion

In this study, we presented a revised version of the testis-specificity score, which relies on data from three transcriptional atlases. Moreover, we present that both supervised and unsupervised machine learning methods on bulk transcriptomic data could help to gain additional understanding of gene expression. We presented here that using dimension reduction methods on different sources of transcriptomic data can be helpful, even without re-evaluation of raw data, to group similarly expressing genes, and find shared features between them. The generalization of the approach requires testing, given that the Drosophila testis is a highly specialised organ. Another interesting question is the scalability of the data, whether the resolution and mapping could be improved further, with additional different transcriptomic data, or with the re-evaluation of raw transcriptomic data.

## Supplementary Information

Below is the link to the electronic supplementary material.

Supplementary Material 1: Additional file S1 The effect of additional data on t-SNE maps. Aggregations in the full dataset were investigated in the dataset without Fly Cell Atlas data. The selected nodes show enrichment in the cell types listed. Pigment, fat and epithelial cell-associated genes show moderate clustering without the fly cell atlas data, and clustered distinctly using the full dataset. Secretory cell and neuron-associated gene nodes are diffuse without the fly cell atlas data. Spermatocyte and spermatid-associated groups remain highly similar with the expansion of the dataset.

Supplementary Material 2: Additional file S2 Heatmap indicating feature importance of XGBoost models predicting testis-specificity. Gain, weight, and cover were investigated in all three models; features represent single lines.

Supplementary Material 3: Additional file S3 Heatmap indicating feature importances of XGBoost models predicting testis-specific transcription factor association. Gain, weight, and cover were investigated in all four models; the features represent single lines.

Supplementary Material 4: Additional file S4 t-SNE data clustering according to DBSCAN. t-SNE graphs represent the groups established by DBSCAN. DBSCAN parameters, established groups, and the number of unclassified nodes are indicated at the top left of the graphs. Subgroups further investigated are listed on the top right (for Figure 5 and Additional Table S3-5). Heatmaps represent the mean testis specificity and mean specificity values of genes in the given group. Groups are represented in columns.

Supplementary Material 5: Additional file S5 Position weight matrices of identified motifs.

Supplementary Material 6: Additional file S5 Collection of motifs’ distribution patterns around the TSS. Diagrams are paired; the first diagram shows the overall distribution, the second shows a breakdown based on strands. Each motif is represented with a general pattern map, a map with genes that have higher than 100 and 1000 expression filter, a map of mip40, can and comr associated genes, and a map of genes filtered based on testis specificity (>0.9, 0.5>, <0, <-0.5).

Supplementary Material 7: Additional file S6 Flow chart represents an overview of the manuscript.

Supplementary Material 8: Additional Table S1 Data processing and testis specificity indices. First sheet contains information about processing literature data for both testis specificity calculation and ML approaches. Second sheet contain information on ML parametrisation and workflow. Third sheet contain the tau scores and z-score testis specificity values that were calculated based on Encode, Fly Atlas, Fly Atlas2.

Supplementary Material 9: Additional Table S2 Features used for machine learning algorithms and source publications.

Supplementary Material 10: Additional Table S3 GO enrichment analysis of DBSCAN clusters with epsilon = 11, minimum samples = 60 (e11, ms60).

Supplementary Material 11: Additional Table S4: GO enrichment analysis of DBSCAN clusters with epsilon = 11, minimum samples = 90 (e11, ms90).

Supplementary Material 12: Additional Table S5GO enrichment analysis of DBSCAN clusters with epsilon = 12, minimum samples = 90 (e12, ms90).

## Data Availability

All data generated during this study are included in this published article and its supplementary information files. Additional information is available: 10.5281/zenodo.18964638.
