# Supplementary figures and images for: Machine learning analysis of Drosophila testis transcriptomic data reveals potential regulatory sequences
Source: BioData Min. 2026 Mar 31;19:37. doi: 10.1186/s13040-026-00552-2 (PMC13162424; doi:10.1186/s13040-026-00552-2)

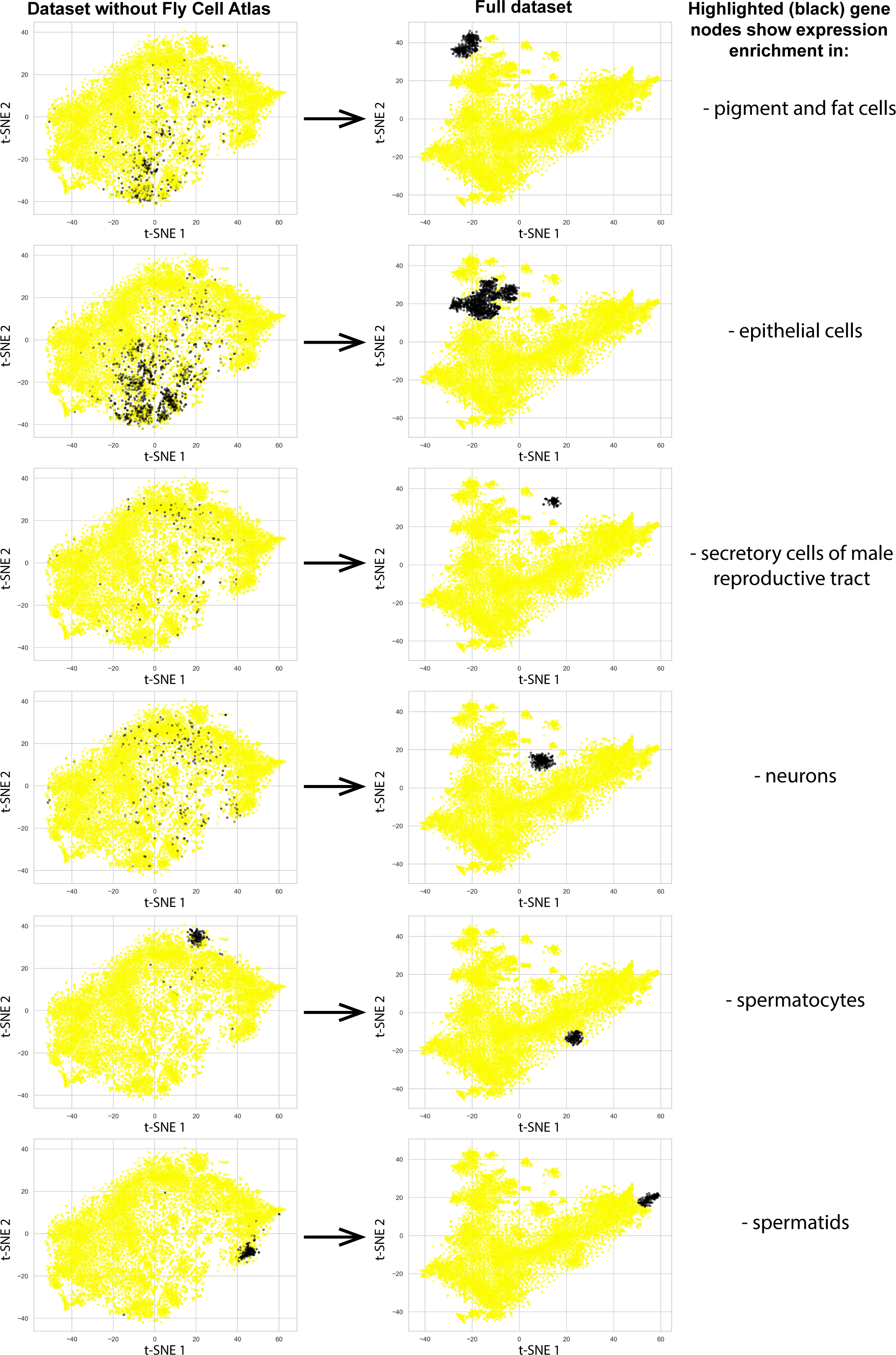

Supplement: Supplementary file 1 — Supplementary Material 1: Additional file S1 The effect of additional data on t-SNE maps. Aggregations in the full dataset were investigated in the dataset without Fly Cell Atlas data. The selected nodes show enrichment in the cell types listed. Pigment, fat and epithelial cell-associated genes show moderate clustering without the fly cell atlas data, and clustered distinctly using the full dataset. Secretory cell and neuron-associated gene nodes are diffuse without the fly cell atlas data. Spermatocyte and spermatid-associated groups remain highly similar with the expansion of the dataset. [file 13040_2026_552_MOESM1_ESM.tif]

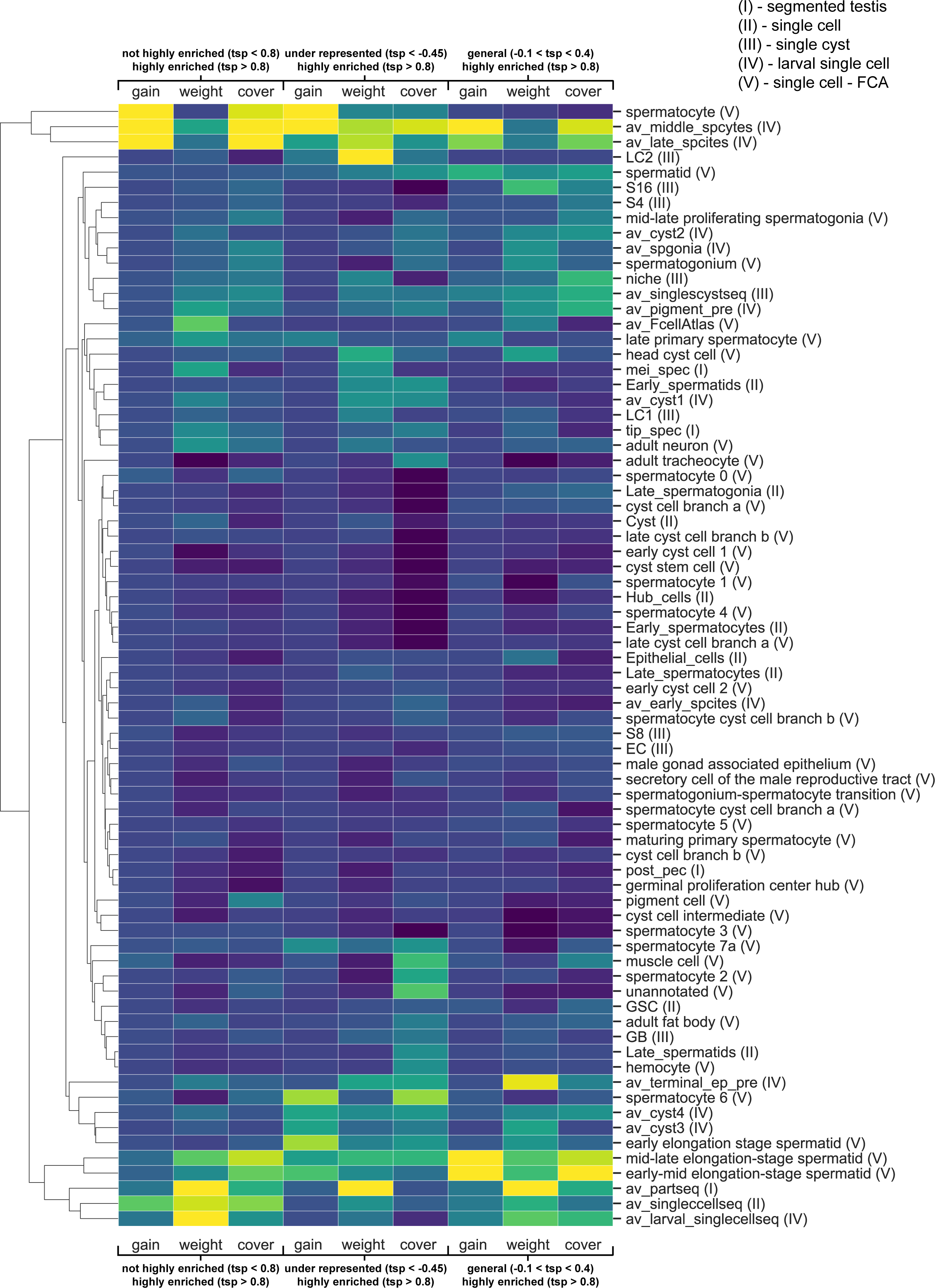

Supplement: Supplementary file 2 — Supplementary Material 2: Additional file S2 Heatmap indicating feature importance of XGBoost models predicting testis-specificity. Gain, weight, and cover were investigated in all three models; features represent single lines. [file 13040_2026_552_MOESM2_ESM.tif]

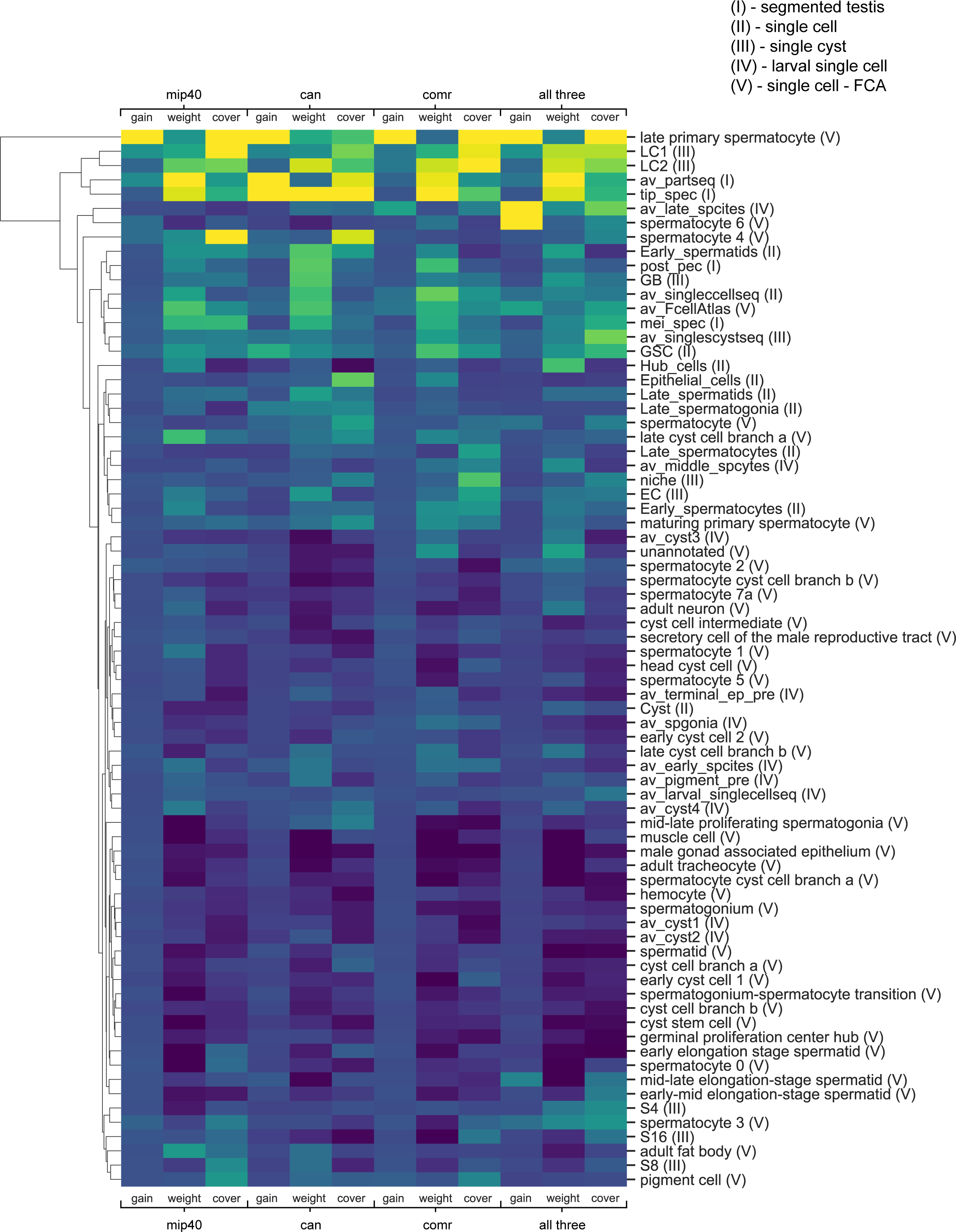

Supplement: Supplementary file 3 — Supplementary Material 3: Additional file S3 Heatmap indicating feature importances of XGBoost models predicting testis-specific transcription factor association. Gain, weight, and cover were investigated in all four models; the features represent single lines. [file 13040_2026_552_MOESM3_ESM.tif]

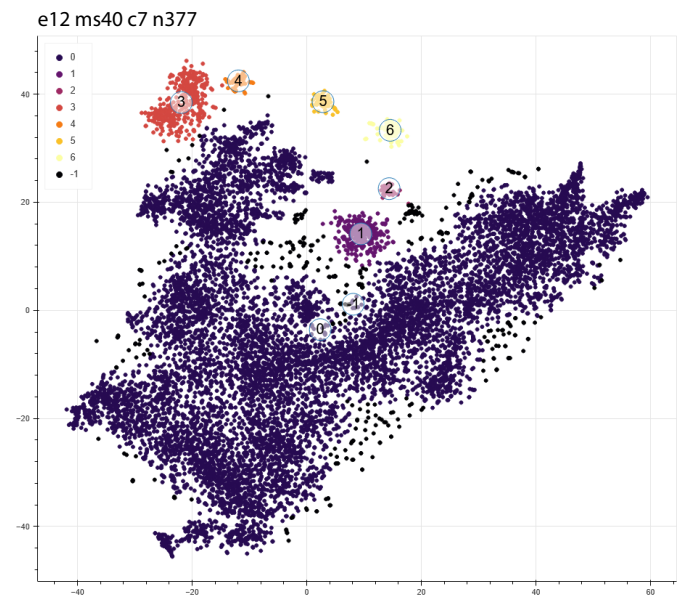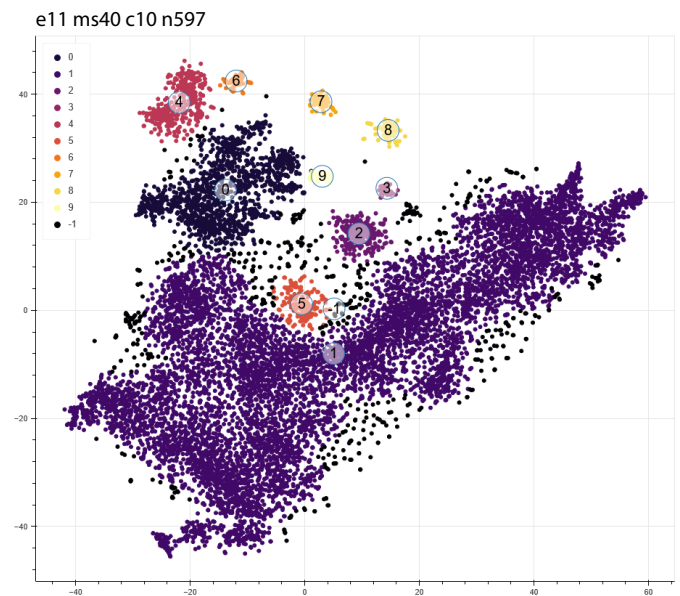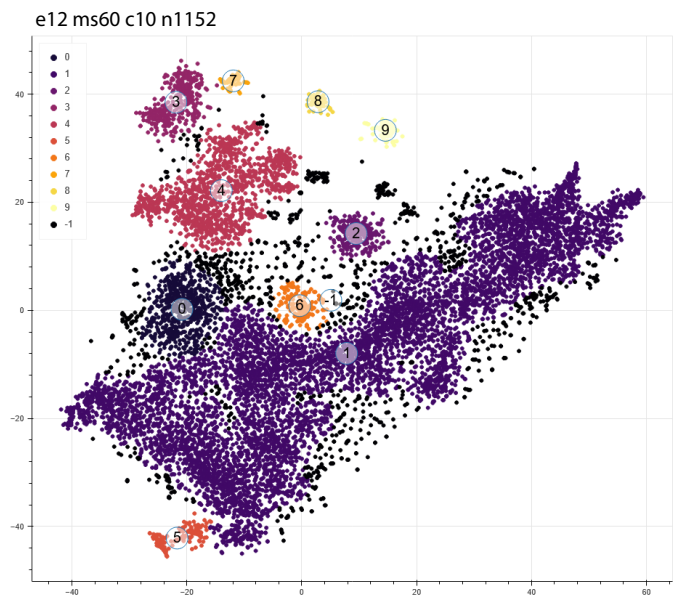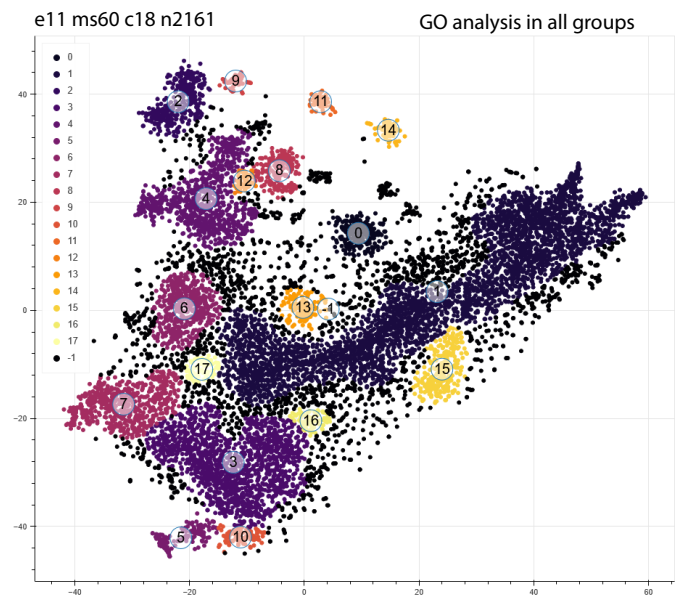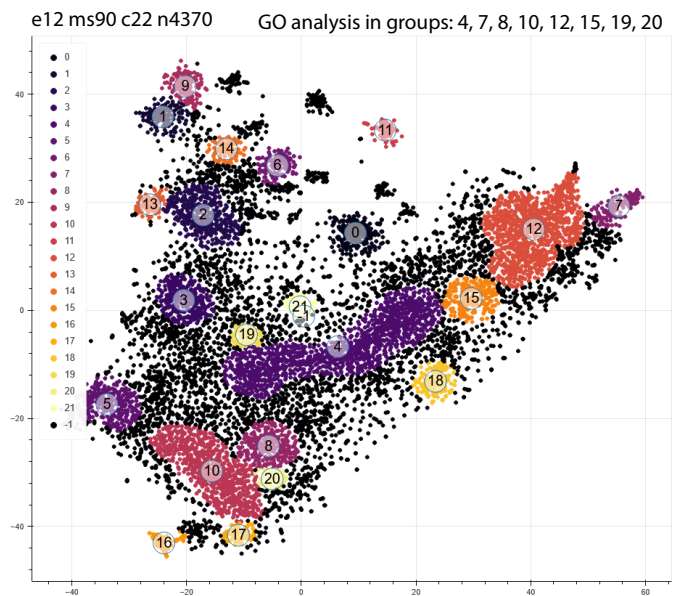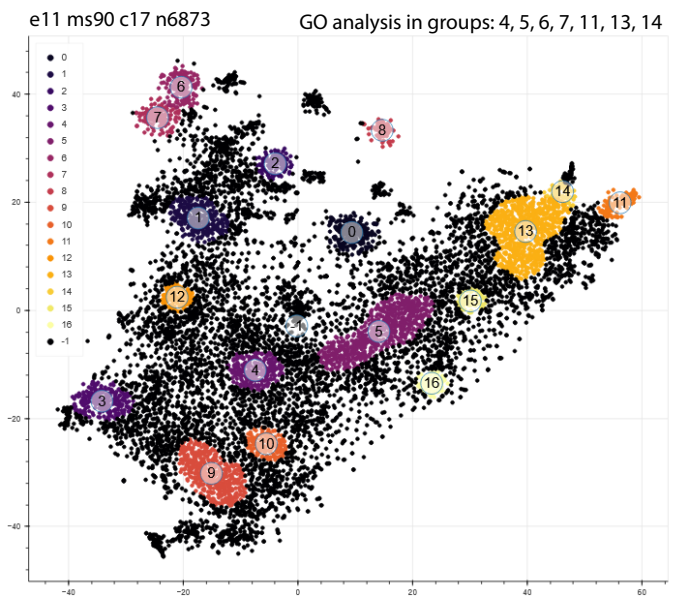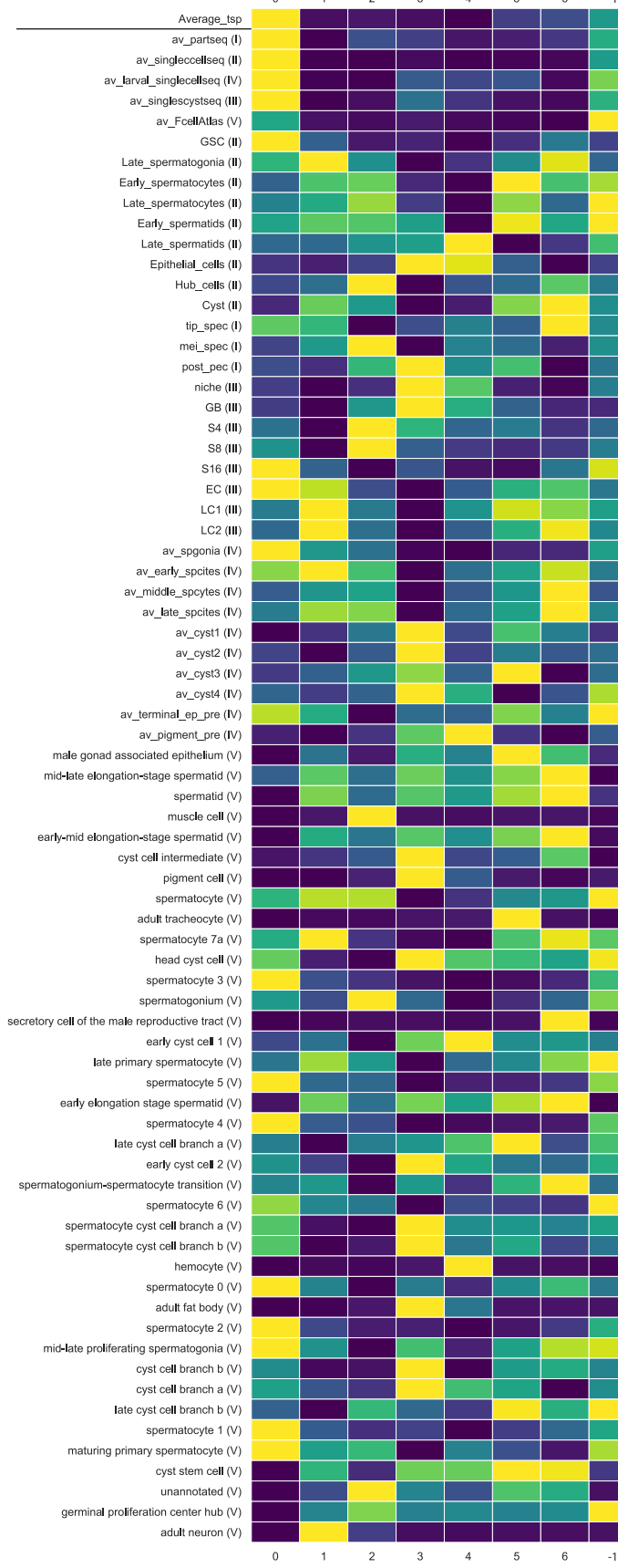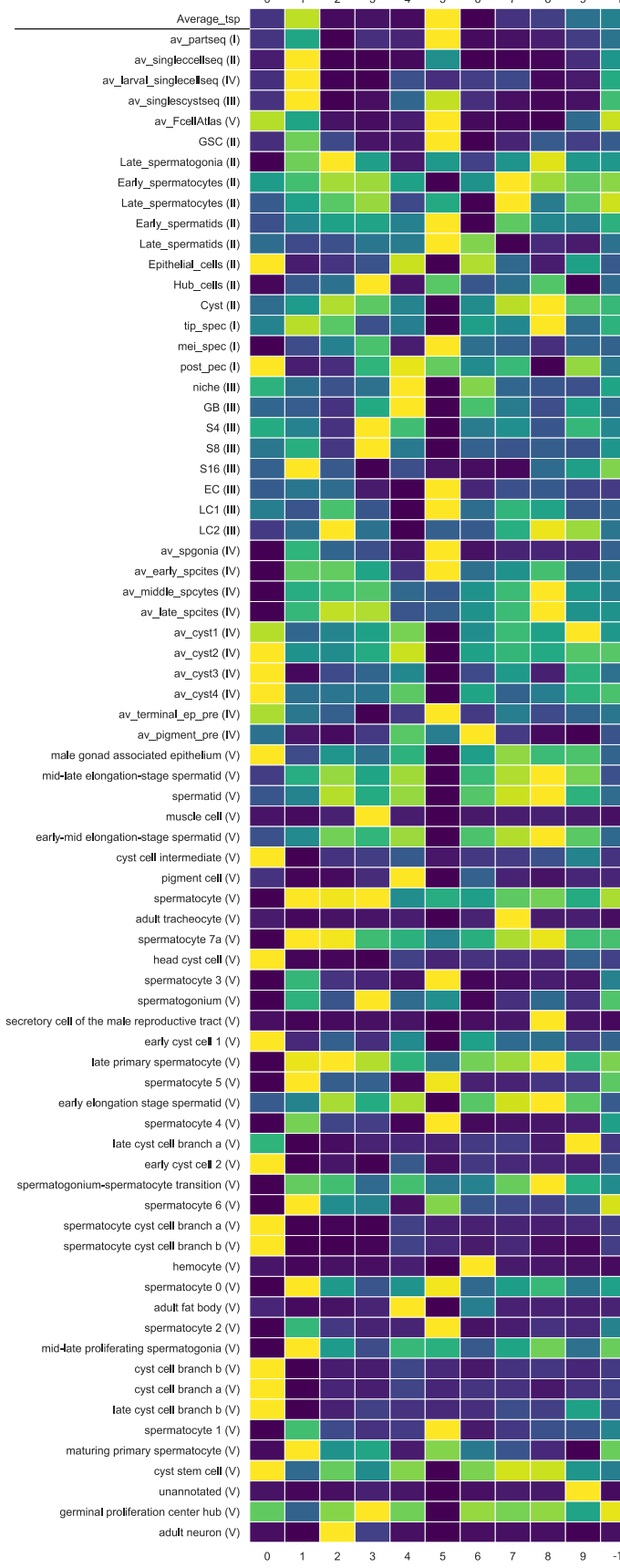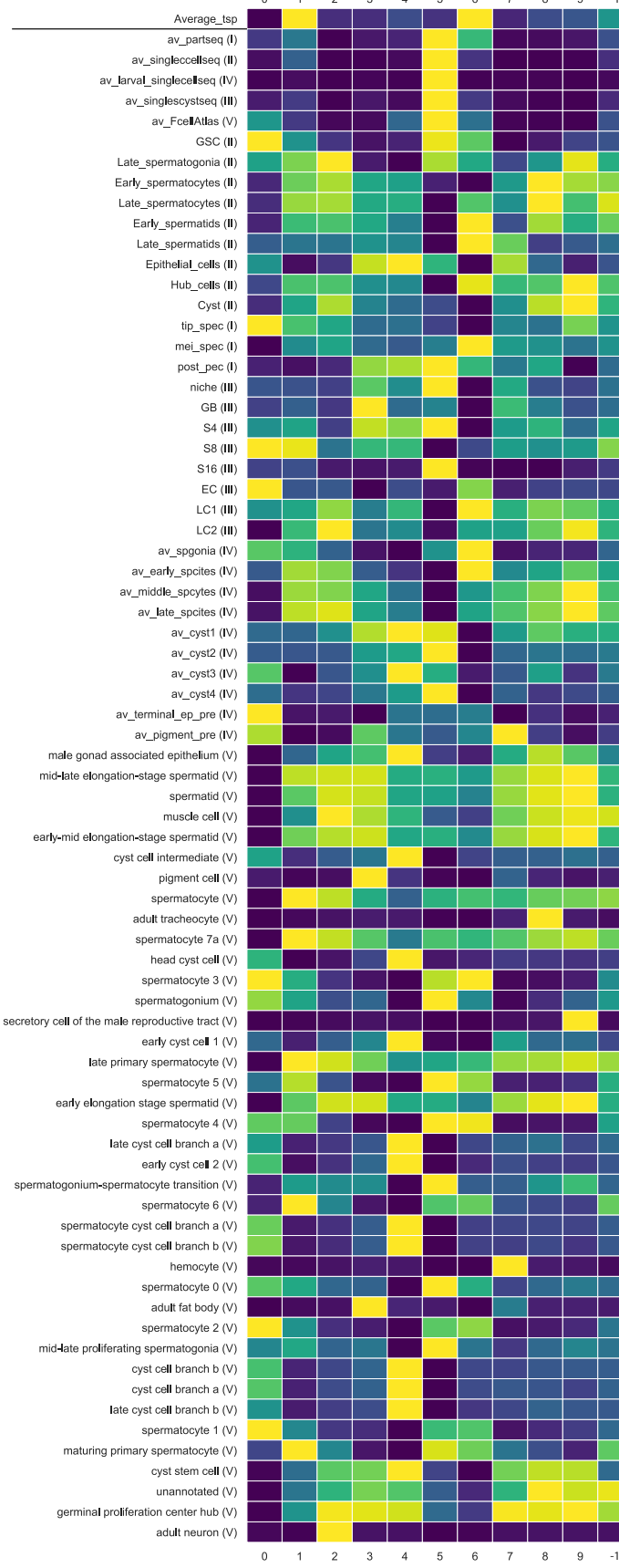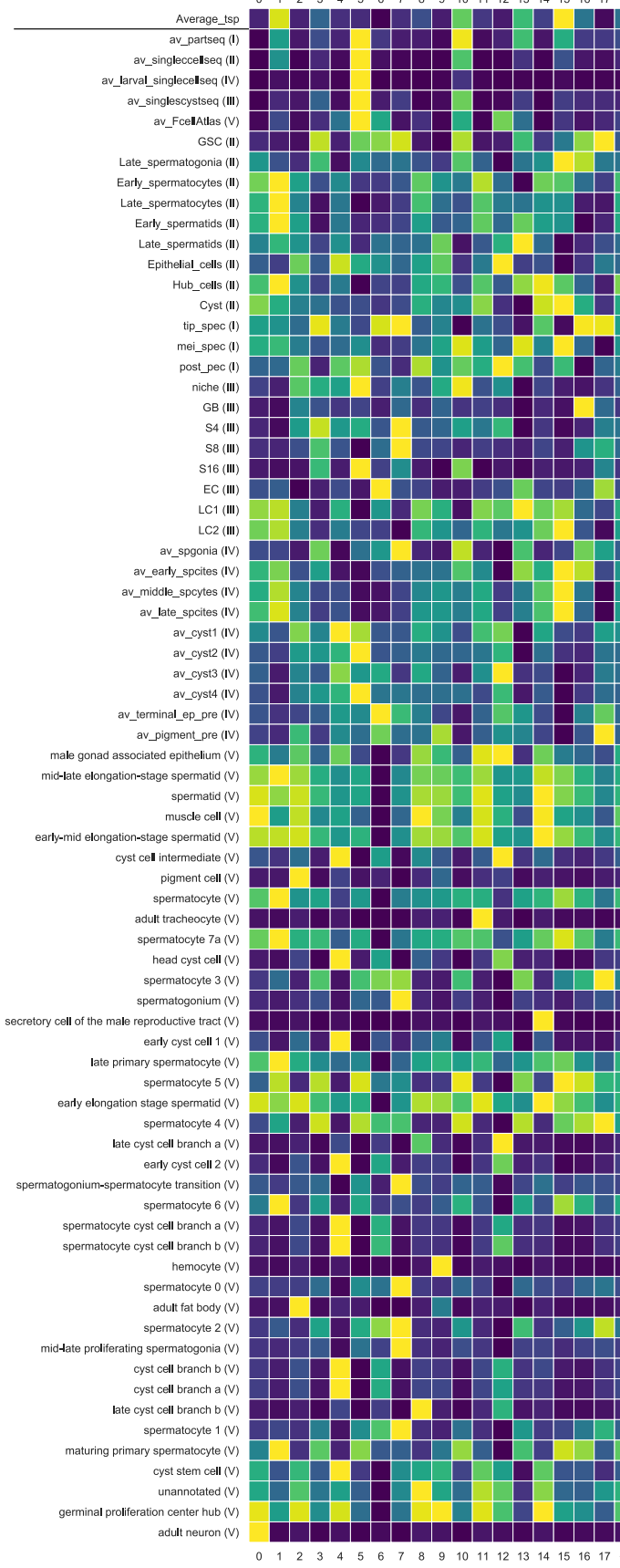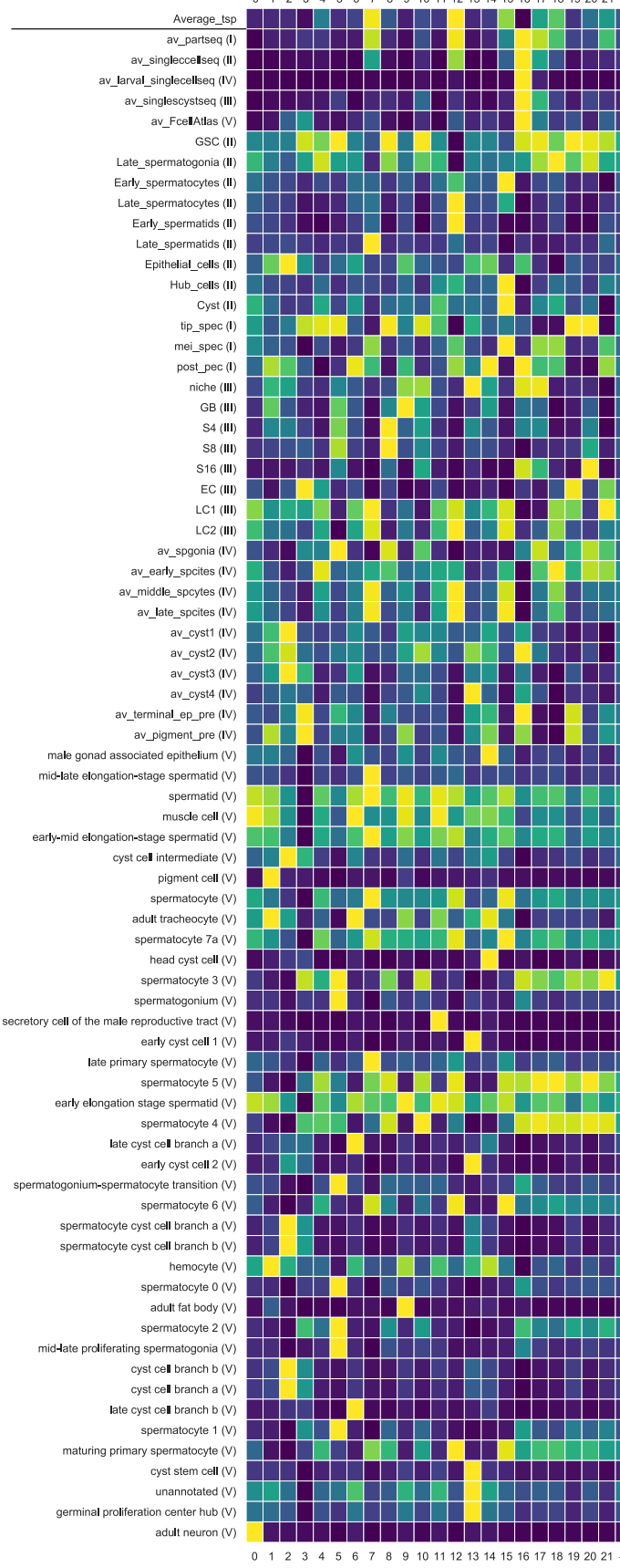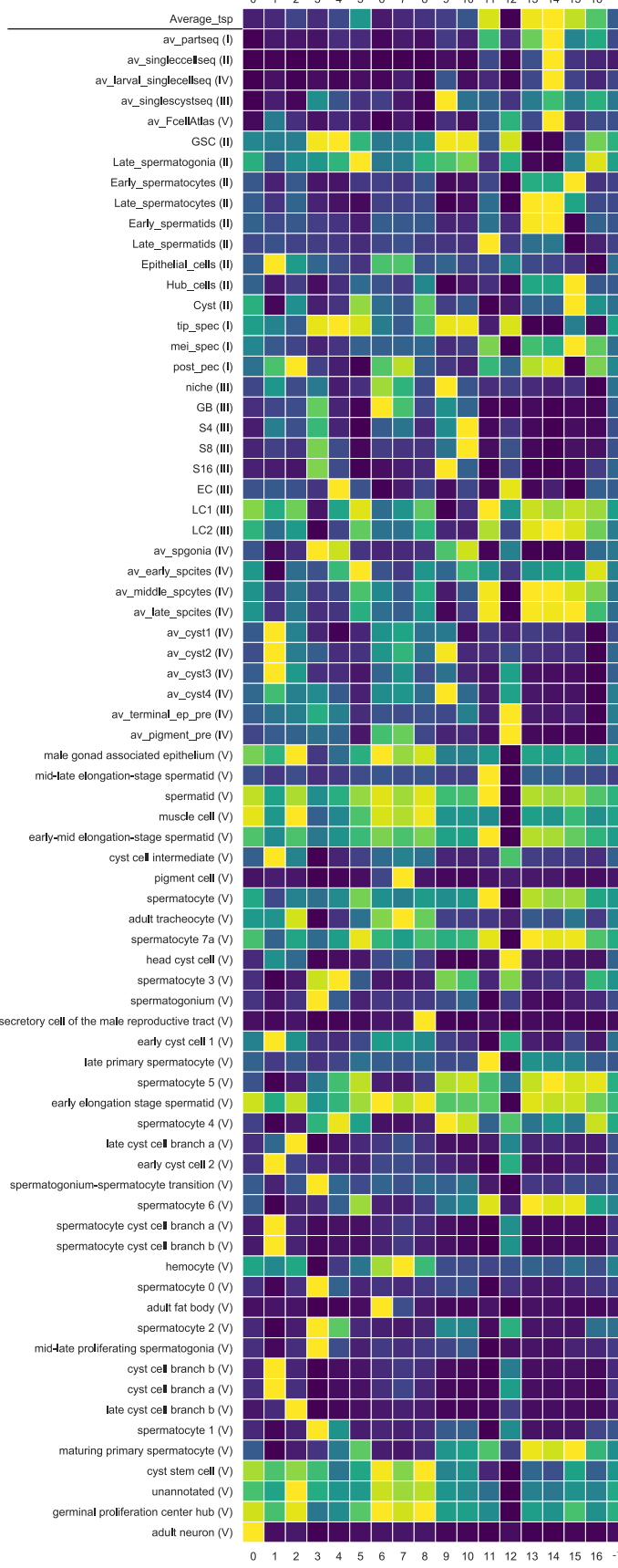

Supplement: Supplementary file 4 — Supplementary Material 4: Additional file S4 t-SNE data clustering according to DBSCAN. t-SNE graphs represent the groups established by DBSCAN. DBSCAN parameters, established groups, and the number of unclassified nodes are indicated at the top left of the graphs. Subgroups further investigated are listed on the top right (for Figure 5 and Additional Table S3-5). Heatmaps represent the mean testis specificity and mean specificity values of genes in the given group. Groups are represented in columns. [file 13040_2026_552_MOESM4_ESM.pdf]

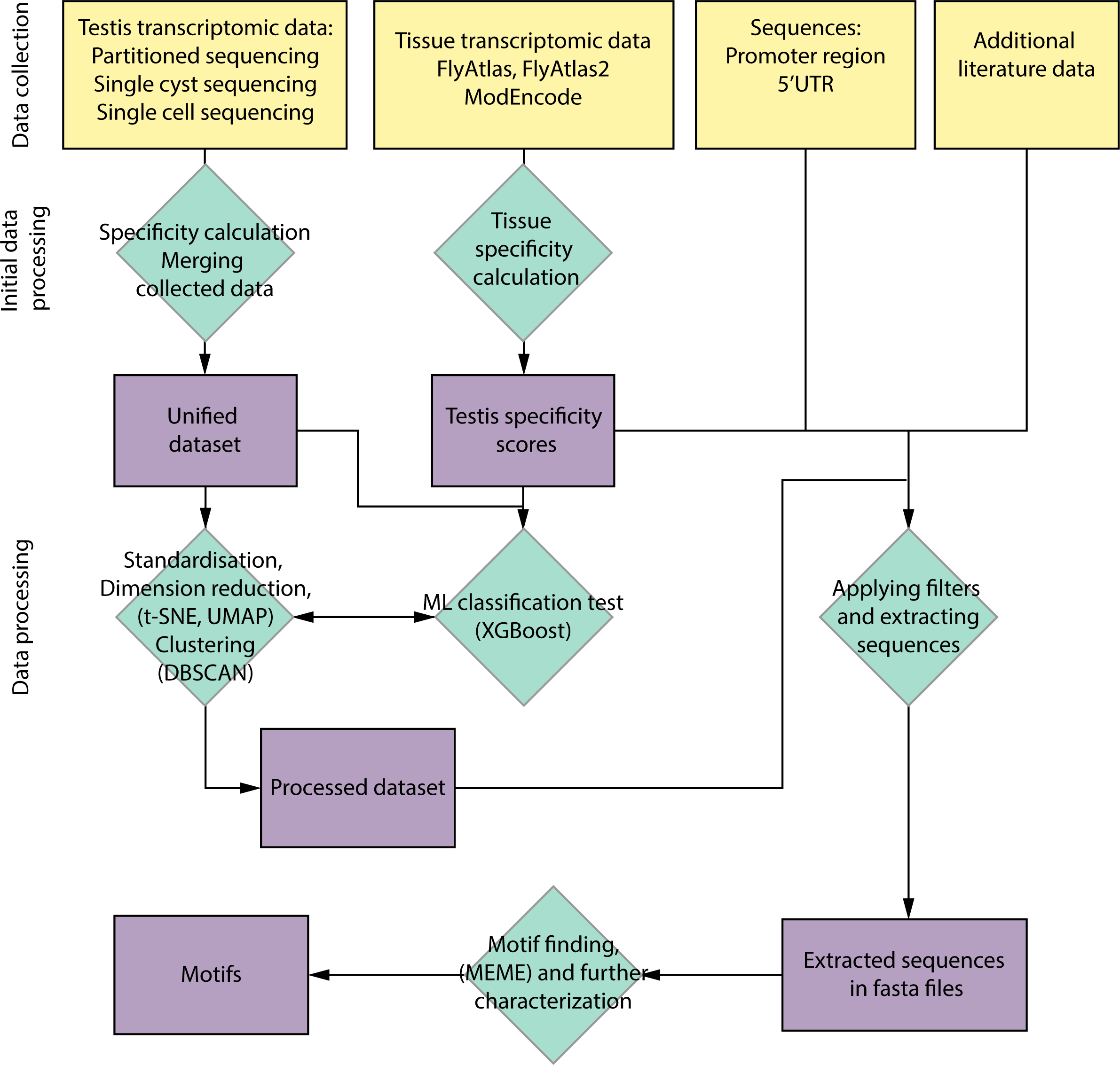

Supplement: Supplementary file 7 — Supplementary Material 7: Additional file S6 Flow chart represents an overview of the manuscript. [file 13040_2026_552_MOESM7_ESM.tif]
