# Supplementary material for: Machine learning analysis of Drosophila testis transcriptomic data reveals potential regulatory sequences
Source: BioData Min. 2026 Mar 31;19:37. doi: 10.1186/s13040-026-00552-2 (PMC13162424; doi:10.1186/s13040-026-00552-2)
Supplement: Supplementary file 6 — Supplementary Material 6: Additional file S5 Collection of motifs’ distribution patterns around the TSS. Diagrams are paired; the first diagram shows the overall distribution, the second shows a breakdown based on strands. Each motif is represented with a general pattern map, a map with genes that have higher than 100 and 1000 expression filter, a map of mip40, can and comr associated genes, and a map of genes filtered based on testis specificity (>0.9, 0.5>, <0, <-0.5). [file 13040_2026_552_MOESM6_ESM.pdf]

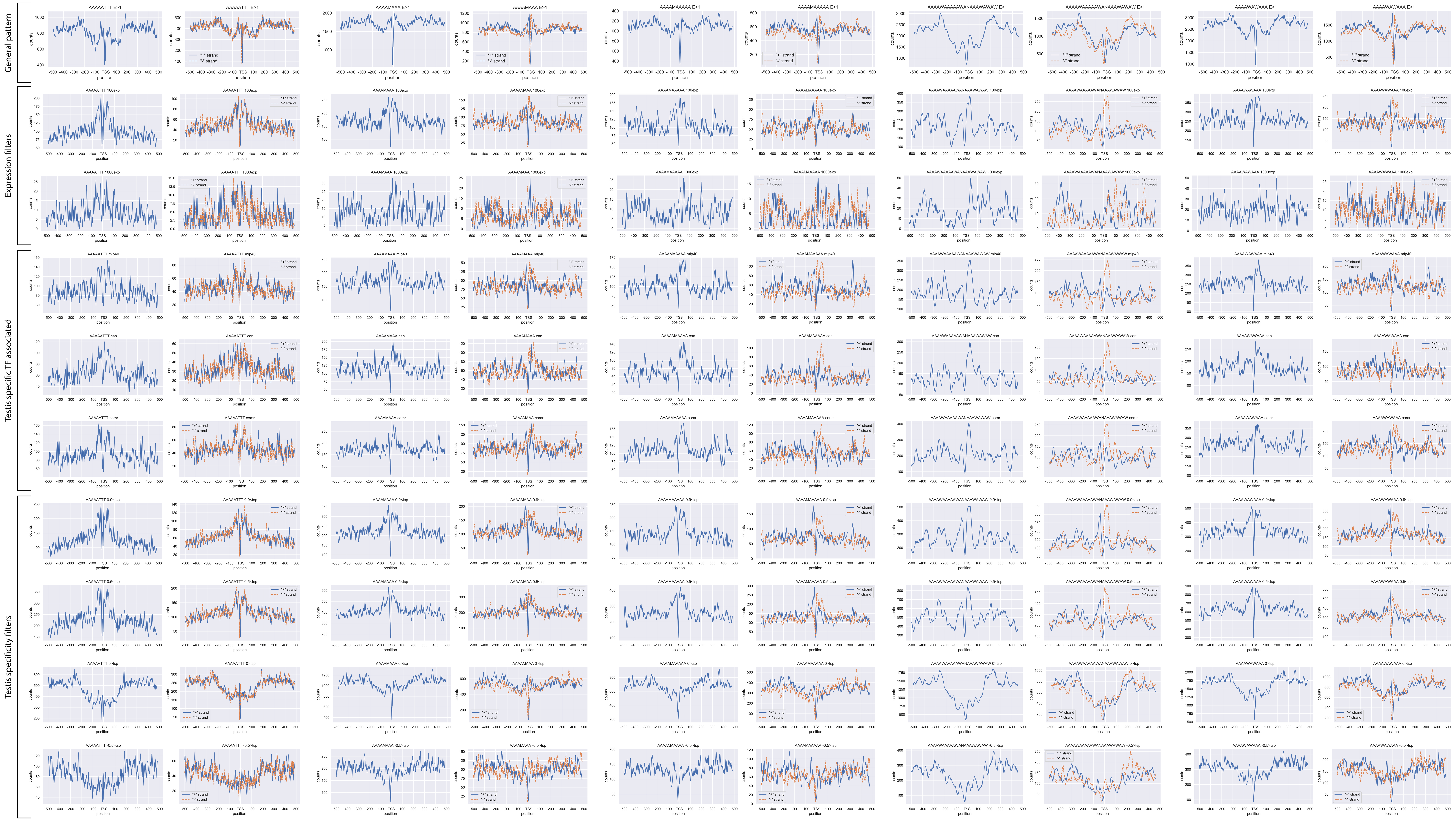
General pattern
Expression filters
Testis specific TF associated
Testis specificity filters

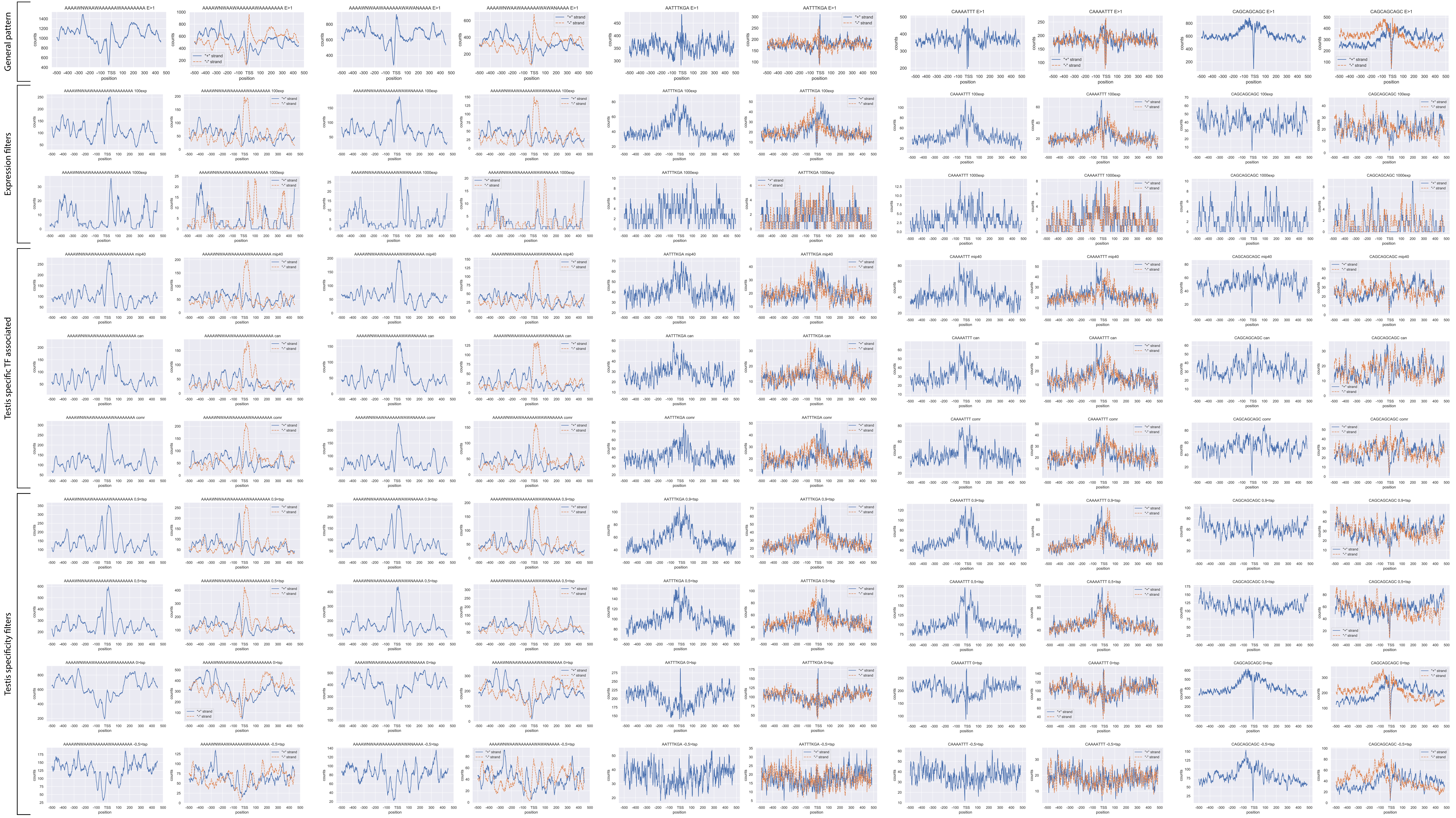
General pattern
Expression filters
Testis specific TF associated
Testis specificity filters
AATTTKGA
CAAAATTT
CAGCAGCAGC
"+" strand
"-" strand
counts
position

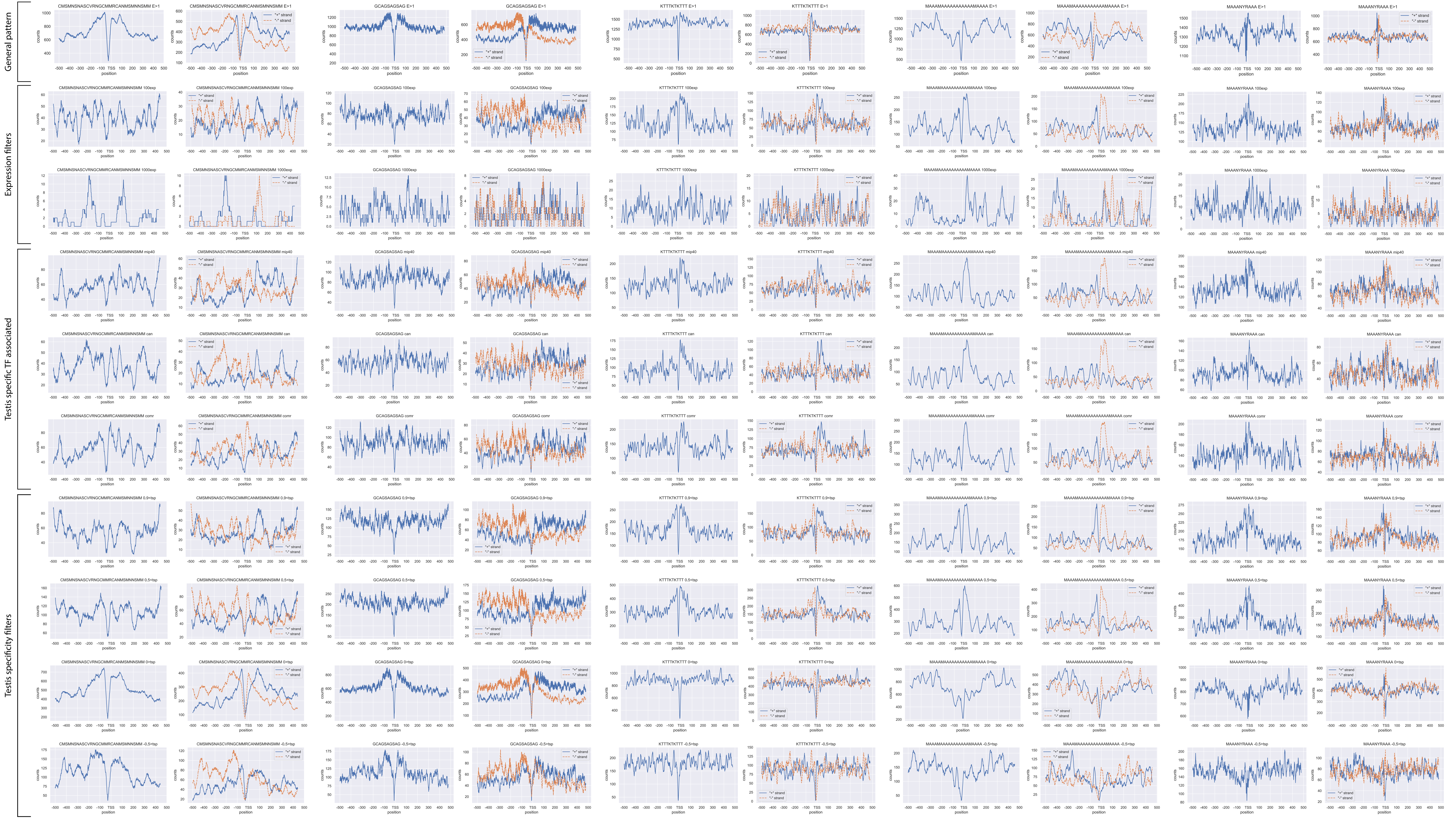
General pattern
Expression filters
Testis specific TF associated
Testis specificity filters
counts
position
"+" strand
"-" strand

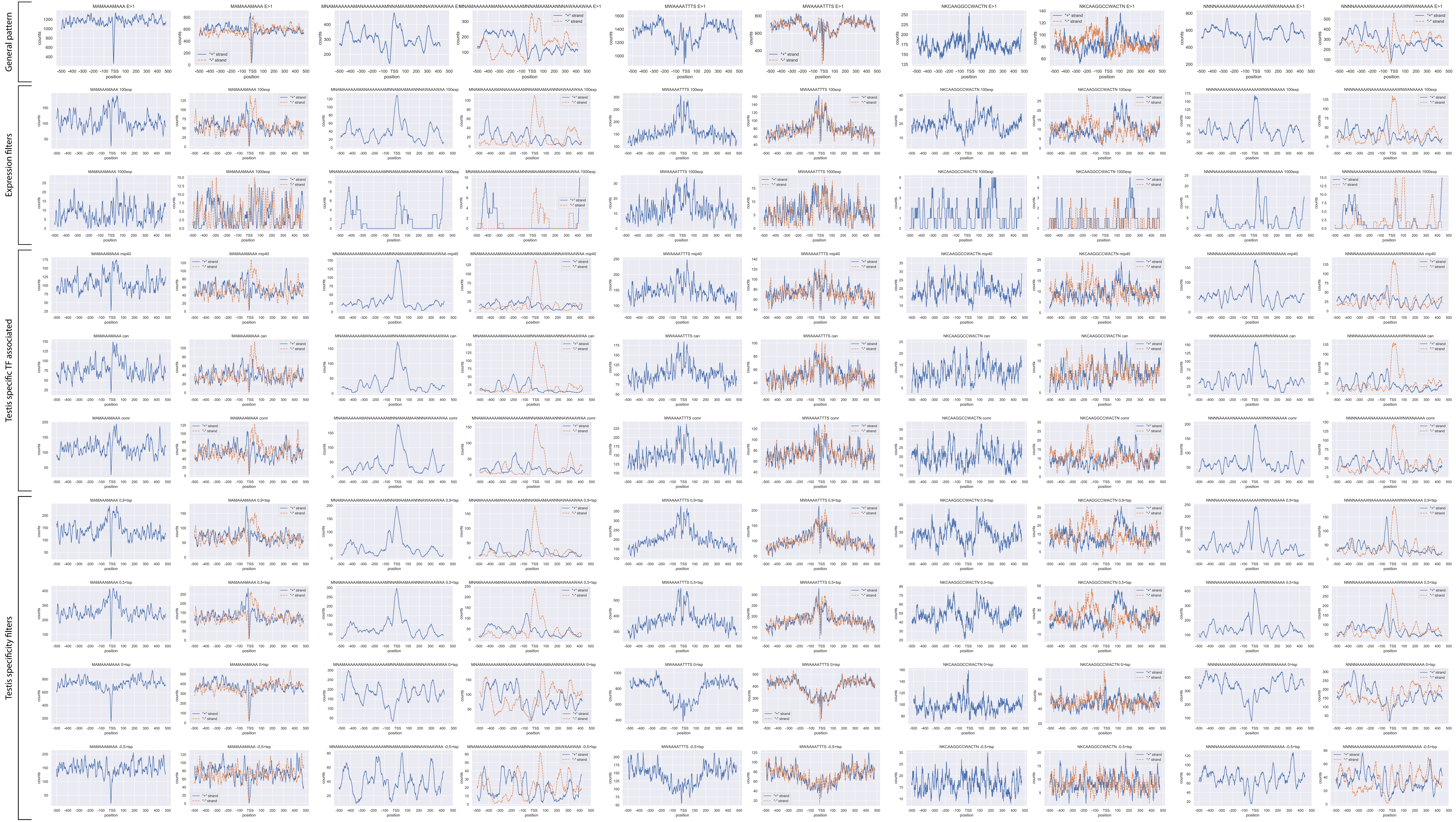
General pattern
Expression filters
Testis specific TF associated
Testis specificity filters
counts
position
TSS
"+" strand
"-" strand

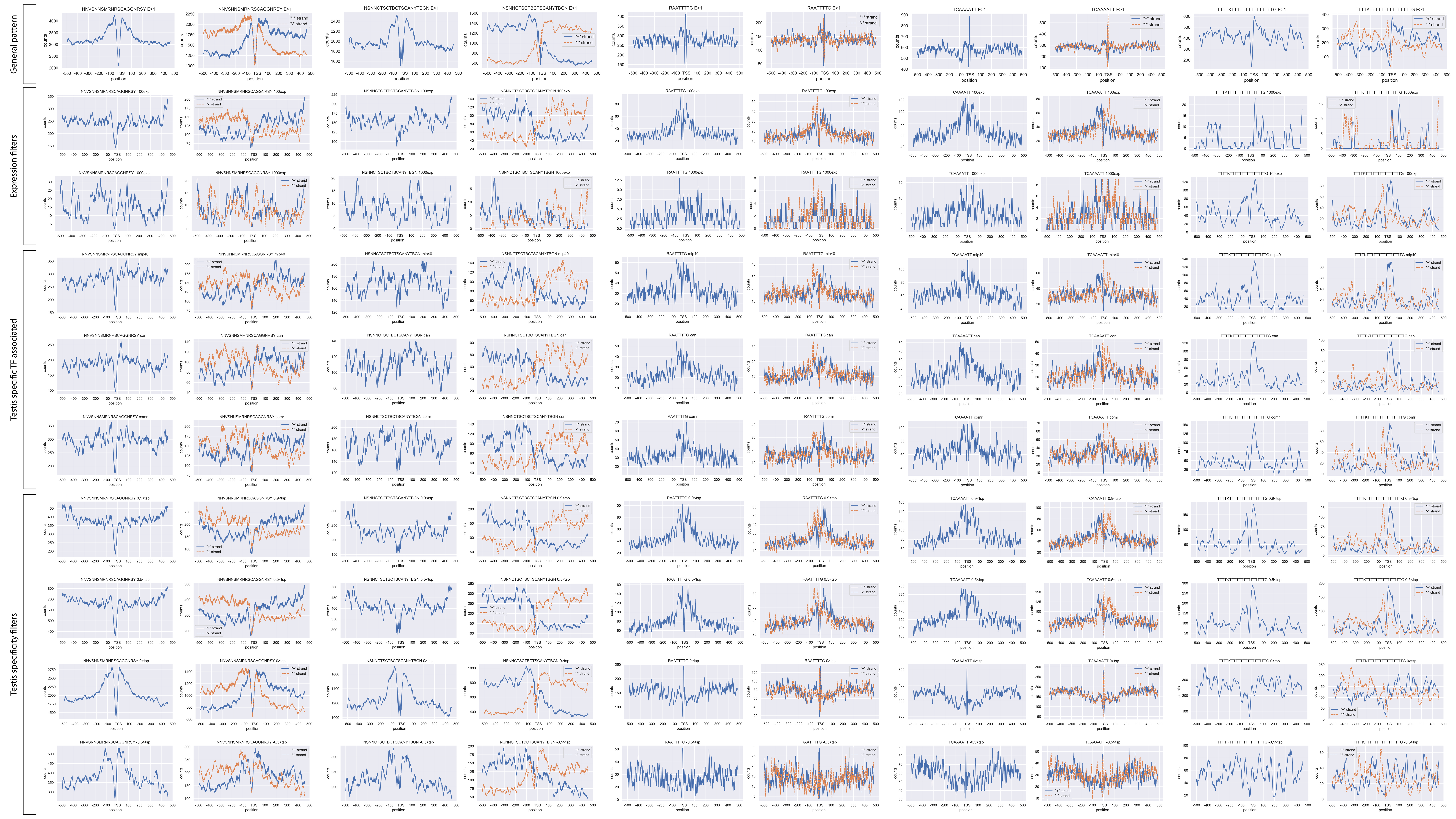
General pattern
Expression filters
Testis specific TF associated
Testis specificity filters
counts
position
"+" strand
"-" strand

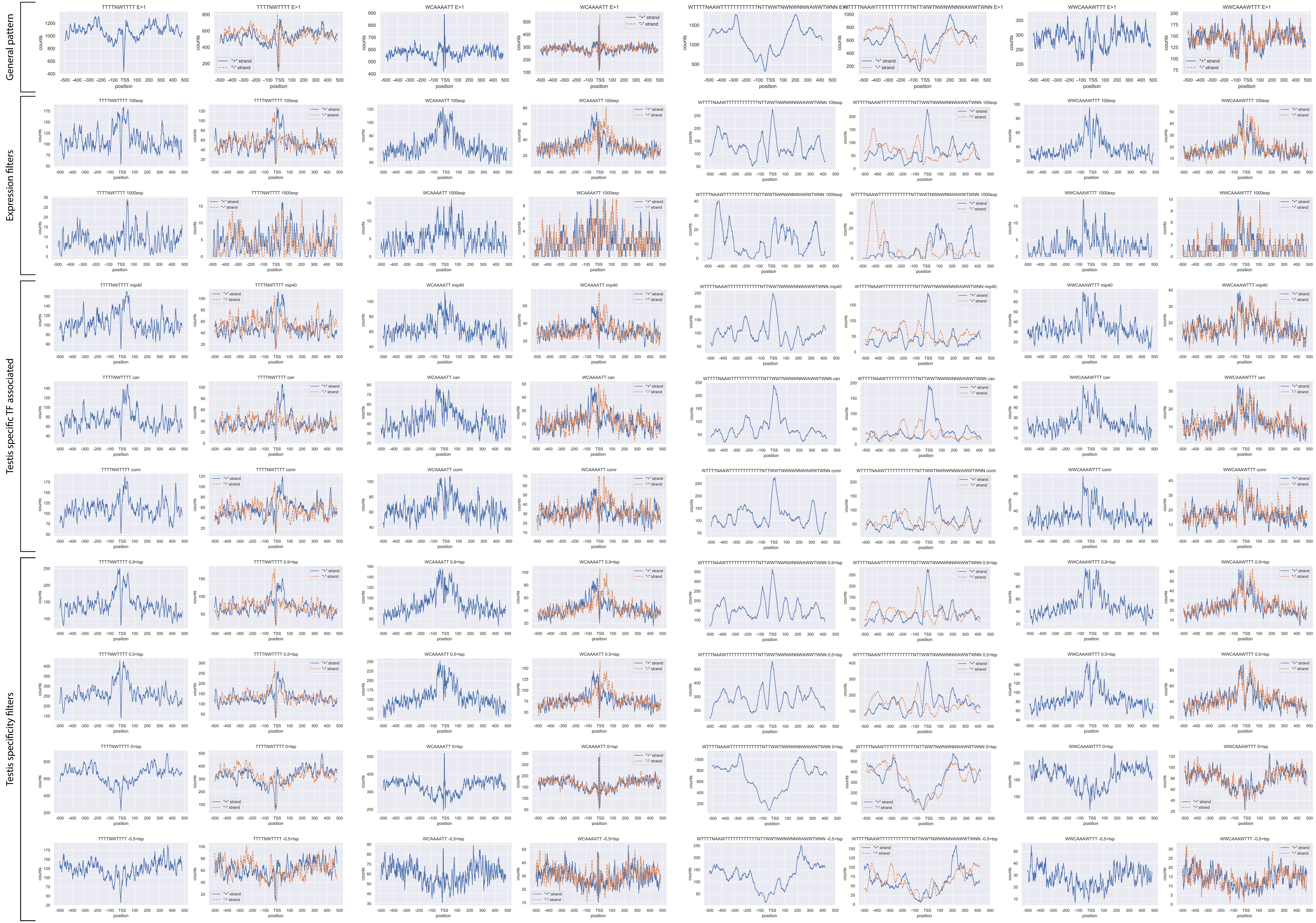
General pattern
Expression filters
Testis specific TF associated
Testis specificity filters
TTTTNWTTTT E>1
WCAAAATT E>1
WWCAAAWTTT E>1
position
counts
"+" strand
"-" strand
